# Comparing PFAS analysis in batch leaching and column leaching tests

**DOI:** 10.1007/s11356-024-35510-0

**Published:** 2024-11-22

**Authors:** Ute Kalbe, Christian Piechotta, Nicole Bandow

**Affiliations:** 1 BAM Federal Institute for Materials Research and Testing: Bundesanstalt für Materialforschung und -prüfung, 12200 Berlin, Germany; 2 Present Address: German Environment Agency, Colditzstraße 34, 12099 Berlin, Germany

**Keywords:** PFAS, Leaching, Column percolation test, Batch test, Eluate pretreatment, Soil

## Abstract

Laboratory leaching tests are tools to assess the mobility of environmental contaminants released from granular materials. Comparative leaching tests were performed using four PFAS-contaminated soils whose concentration patterns of 10 selected perfluoroalkyl and polyfluoroalkyl substances (PFAS) differed due to the two types of contamination sources. This study aimed to evaluate the equivalence of two usual laboratory-scale leaching test procedures, batch and column percolation tests, at liquid-to-solid ratios (L/S) of 2 l/kg, which is the current practice within the German assessment framework, and 10 l/kg (relevant for some EU regulations such as the landfill directive). The differences between the replicates of leaching tests investigating PFAS were smaller for column percolation tests than for batch tests, probably mainly due to the greater sample size and the better representativeness of the sample portion analyzed. It was observed that batch tests overestimate the release of shorter-chain PFAS, whereby the effect was greater with carboxylic than with sulfonic acids. Currently, the limits of detection of analyses given by the DIN standard with regard to PFCA and PFSA in soils are partly not sufficient to detect very low contents, whereas the detection of selected PFCA and PFSA in eluates is more sensitive, in accordance with the available standards. This results in limitations when calculating mass balances.

**Supplementary Information:**

The online version contains supplementary material available at 10.1007/s11356-024-35510-0.

## Background

Perfluoroalkyl and polyfluoroalkyl substances (PFAS) are widely used due to their ability to provide surfaces with beneficial properties, such as repellency to water, grease, and dirt. Fields of applications include their use as agents in firefighting foams, paper products, galvanic processes, outdoor clothing, and cosmetics (Posner 2012). The intensive use of such compounds leads to their widespread transfer into the environment, either during production and industrial use or by release from consumer products. Since PFAS are very persistent substances, they are hardly broken down in the environment (Ahrens and Bundschuh 2014). They have been detected in surface waters, soils, groundwater, drinking water, sewage sludge, and biota (Ahrens and Bundschuh 2014; Kotthoff et al. 2020). The use of the most prominent PFAS was restricted or banned by inclusion of perfluorooctane sulfonic acid (PFOS), its salts, and perfluorooctane sulfonyl fluoride (POSF) in 2009 in Annex B of the Stockholm Convention on Persistent Organic Pollutants and of perfluoro-*n*-octanoic acid (PFOA) including its salts and precursors in Annex A 2019 (United Nations Environment Progamme 2004; Fiedler et al. 2019; Brunn et al. 2023). POSF is used in the production of PFOS. Longer transition periods were granted only for selected uses such as medical implants and fire extinguishing foams. In Europe, the ban on the manufacture and marketing of PFOA including its salts and precursors came into effect in 2020 (REACH, Commission Regulation (EU) 2020/748 (European Commission 2020)). The ban has not led to a decrease in the use of PFAS, but rather to a substitution by other PFAS. This includes on the one hand smaller, more mobile ones with less carbon atoms than PFOS or PFOA and on the other hand, fluorotelomers which may degrade to PFAS such as PFOS and PFOA (Butt et al. 2014). A proposal to restrict the use of the whole group in the EU is currently under review (ECHA 2023).

Wastewater treatment plants are a hotspot of contamination of surface water; because PFAS are not effectively removed from wastewater originating from industry and domestic sources, a portion of the PFAS with longer chain lengths may adsorb to the sludge (Chen et al. 2018; Coggan et al. 2019; Meegoda et al. 2020; Link et al. 2024). Soil is particularly contaminated with PFAS that are released when used as firefighting agents, especially at training sites (Baduel et al. 2015; Anderson et al. 2016; Schaefer et al. 2023), and by the deposition of contaminated sludge (Kotthoff et al. 2020; Röhler et al. 2021; Bierbaum et al. 2023). Shorter perfluoroalkyl carboxylic acids, perfluoroalkyl sulfonic acids, and volatile fluorotelomers can also be transported through the air to more remote areas (Butt et al. 2014; Wang et al. 2015; Washington et al. 2019). Uptake in plants (Blaine et al. 2013), such as vegetables, or by leaching from the soil to the groundwater, which can be an important source of drinking water production (Zareitalabad et al. 2013), can then introduce PFAS to the human food chain. The transport of PFAS through the soil depends mainly on their chain lengths. Shorter PFAS are more mobile due to their polarity and reach the groundwater faster (Stahl et al. 2013; Li et al. 2018; Nguyen et al. 2020). It has been found that matrix characteristics of soil (e.g., organic matter content) and the concentration of PFAS also play an important role (Guelfo and Higgins 2013).

Groundwater-monitoring wells are a tool to investigate the transfer of dissolved PFAS and have been installed particularly on contaminated sites (Høisæter et al. 2019). Investigations to study the mobility of PFAS using lysimeters are quite rare, but have proven the faster transfer of short-chain molecules and the long persistence of PFAS (Stahl et al. 2013; McLachlan et al. 2019). Lysimeter studies are not used as standard tests, due to their long-term scale of several years. The transfer of PFAS from soil to groundwater has been modeled, whereby most models have some limitations described elsewhere (e.g., McLachlan et al. 2019; Arcadis U.S. 2020). However, sorption parameters, such as *K*_oc_ and *K*_*d*_, have not always been determined for all substances, or they vary widely in the literature, probably due to soil-specific characteristics and the calculation methods (Guelfo and Higgins 2013; Milinovic et al. 2015; Li et al. 2018; Guelfo et al. 2020; Guo et al. 2020; ITRC 2020; Van Glubt et al. 2021). Therefore, standardized leaching tests on a laboratory scale are well-established tools and can serve as alternative assessment methods. Column percolation tests and batch tests are leaching procedures suitable to investigate the mobility of soil contaminants, whereby many procedures have been elaborated worldwide (Kalbe et al. 2008; López Meza et al. 2008; Grathwohl and Susset 2009; Garrabrants et al. 2012; Naka et al. 2016) and are generally based on the same principles. European batch test standards, in particular the EN 12457 series (EN 12457–2 2002; EN 12457–4 2002), are widely applied to solid matrices. However, the liquid/solid separation step, as it has been specified so far, is not suited for investigating organic contaminants. The requirements for the analysis of organic substances in eluates are usually stricter and more laborious than for inorganic substances. The European column percolation test standard EN 14405 (2017) has recently been updated and now contains appropriate specifications for preparing the eluates to investigate organic substances. However, validation data for organic substances are not available. The international leaching standards for soil (e.g., ISO 21268 series (ISO 21268–1 2019; ISO 21268–2 2019; ISO 21268–3 2019)) were elaborated based on the European waste standards, but have been substantially improved over the years, considering specific soil issues. They now comprise appropriate specifications for the investigation of organic substances. However, ISO standards usually must not be transferred into national regulations, as is the case for harmonized CEN standards within the European Union. In the USA, substantial work has been done developing leaching tests within the Leaching Environmental Assessment Framework (LEAF), which comprises column and batch test protocols, among others (US EPA 1314 and US EPA 1316 (US EPA 2019a)).

Batch tests are known to be easier to perform because of the equipment required, and they enable the investigation of leaching behavior at specified liquid-to-solid ratios (L/S) under the assumption of equilibrium conditions. Batch tests can be disadvantageous compared with column tests because more steps are required to prepare the eluates for analysis, which may lead to losses or cross-contamination. Column percolation tests collect several fractions at certain L/S and therefore provide additional information on time-dependent leaching behavior (Kalbe et al. 2008). Whereas column tests can serve as a tool for the basic characterization of granular materials, batch tests are designed for compliance testing, i.e., comparison with limit values and routine testing of previously characterized materials. Column percolation experiments are thought to provide results closer to field conditions, even if the flow conditions are mostly not comparable to those in the field (Kalbe et al. 2008; López Meza et al. 2008; Grathwohl 2014; Banzhaf and Hebig 2016). Moreover, column tests represent dynamic equilibrium conditions. Whether the test results of both test types at the same L/S are comparable may depend on the substances under investigation; this has to be proven for every class of compounds in combination with variations in the matrices.

The general suitability of column percolation tests for investigating soil contaminated with PFAS has been studied before (Kalbe et al. 2014; Banzhaf and Hebig 2016). To the best of our knowledge, the suitability of various available leaching procedures for this class of organic substances has not yet been compared with the focus on risk assessment, and little is known about the reliability of leaching tests for investigating soils contaminated with PFAS. Therefore, it is urgent to establish leaching test procedures with suitable test conditions to investigate PFAS that can be integrated in legal regulations.

The influence of drying as a sample preparation step was investigated for PFAS using batch leaching tests (Lange et al. 2020), leading the authors of this study to the conclusion that drying samples increases their PFAS concentration. The effect was greater for perfluoroalkyl carboxylic acids (PFCA) than for perfluoroalkyl sulfonic acids (PFSA).

The focus of this study was not on assessing the levels of PFAS contamination on the sites from which the samples were taken, but on comparing the equivalence of the results of the two leaching test procedures for differing soil characteristics and contamination patterns. The aim of the study presented here was to investigate if batch and column leaching tests are comparable for a selection of PFAS and to obtain more information on the influence of eluate pretreatment before analysis (e.g., filtration). The available standard for solid matter (DIN 38414–14 2011) currently includes 10 substances only (seven PFCA and three PFSA). Additionally, there are only limit values available for a few well-studied substances. Therefore, the selection of the investigated PFAS was limited to these frequently analyzed species. These substances were present in sufficient concentrations in the selected soil samples.

## Materials and methods

### Soil samples

The study used four soil samples originating from areas with known PFAS contamination. Soil samples TE (Tempelhof) and TX (Tegel) are from former airports in Berlin/Germany contaminated with PFAS by the use of fire-extinguishing foams. The authors sampled the TE soil from a pile of excavated soil during remediation work at the fire training site of the former airport. The TX sample was taken as a composite sample of excavated soil from a contaminated spot previously identified after the closure of the airport.

Soil samples RA1 and RA2 are from agriculturally used areas in the environment of Rastatt (Baden-Württemberg, Germany), where the contamination probably resulted from the use of compost-containing sludge residues from the paper industry (Kotthoff et al. 2020). The sample RA1 was a reserve sample provided by a soil protection authority responsible for the area of the contaminated site. A company dealing with remediation measures in the same area delivered the RA2 sample. Both soil materials are composite samples from a grid sampling. Thereby, the samples were obtained using PFAS-free sampling equipment and storage containers made from tinplate.

All soil samples were air-dried (ambient air, < 30 °C), which was applied to facilitate the subsequent processing of the samples in terms of flowability and to stabilize them for storage. The samples were then homogenized using a gyro-wheel mixer and divided into sub-samples of appropriate mass for the tests and analyses, using a cross-riffling procedure (van der Veen and Nater 1993) and rotating dividers to ensure the representativeness of the samples. When preparing the samples, care was taken to ensure that only PFAS-free equipment was used (sieves, sample dividers, containers etc.). The samples were stored in representative subsamples in 250-ml brown glass bottles using aluminum foil as an insert in the lid at 4 °C in a dark climate room before performing the leaching tests.

A representative sub-sample of about 10 g of each soil was ground to < 250 µm using a mixer mill (Retsch MM200, balls and 10-ml grinding bowl made of zirconium oxide ceramic) for the content analyses.

### Basic characterization of soil samples

The residual moisture in the samples was determined in accordance with DIN ISO 11465 (1996) after constant mass was achieved by drying at 105 °C. Loss on ignition (DIN 18128 2002) was used to determine each sample’s organic carbon content. The dry sample was heated at 550 °C in a muffle furnace until constant mass was achieved. Carbonate content was determined in accordance with DIN ISO 10693 (2014), using hydrochloric acid to transform carbonate into carbon dioxide and then using a pressure sensor to measure the amount of CO_2_ formed. The total carbon content (TC) was measured using a CS-800 Carbon Sulfur Analyzer (Eltra) and detected via the IR absorption method. Particle density was obtained using a He-pycnometer in accordance with DIN 66137–2 (2019). The pH value (DIN ISO 10390 2005) and electrical conductivity (DIN EN 27888 1993) were measured after preparation of an aqueous slurry. All parameters were obtained from at least two independently performed measurements.

### PFAS analysis

Ten selected individual compounds in solid soil samples (see Table 1) were analyzed in accordance with DIN 38414–14 (2011): soils were extracted after the addition of ^13^C-labeled internal standard solution with methanol. For the 10 targeted PFAS (Table 1), four ^13^C-labeled internal standards were used in accordance with the German DIN standard 38414–14 (^13^C-PFOS: PFBS, PFHxS, PFOS; ^13^C-PFOA: PFHpA, PFOA, PFNA, PFDA; ^13^C-PFHxA: PFPeA, PFHxA; ^13^C-PFBA: PFBA).

**Table 1:** Overview of the selected PFCA and PFSA (identical to the current selection in the analytical standards) including information an CAS number, sum formula, water solubility, and molecular mass

	Name	CAS number	Sum formula	Water solubility^a^ (mg/l)	Molecular mass (g/mol)
PFBA	Perfluorobutanoic acid	375–22-4	C_4_HO_2_F_7_	1373	214.04
PFPeA	Perfluoropentanoic acid	2706–90-3	C_5_HO_2_F_9_	196	264.05
PFHxA	Perfluorohexanoic acid	307–24-4	C_6_HO_2_F_11_	27.1	314.05
PFHpA	Perfluoroheptanoic acid	375–85-9	C_7_HO_2_F_13_	3.65	364.06
PFOA	Perfluorooctanoic acid	335–67-1	C_8_HO_2_F_15_	0.48	414.07
PFNA	Perfluorononanoic acid	375–95-1	C_9_HO_2_F_17_	0.062	464.08
PFDA	Perfluorodecanoic acid	335–76-2	C_10_HO_2_F_19_	0.008	514.08
PFBS	Perfluorobutane sulfonic acid	375–73-5	C_4_HO_3_F_9_S	344	300.10
PFHxS	Perfluorohexane sulfonic acid	355–46-4	C_6_HO_3_F_13_S	0.104	400.11
PFOS	Perfluoroctane sulfonic acid	1763–23-1	C_8_HO_3_F_17_ S	0.104	500.13

^a^EPI Suite prediction using *K*_OW_ (US EPA 2019b)

Aqueous eluate samples were analyzed for the same selected substances in accordance with DIN 38407–42 (2011) and after 1:1 dilution. The dilution step is practiced to identify possible ion suppression in the ESI (electrospray ionization) source caused by matrix components like humic substances. Concentrations were determined using LC–MS/MS operated in the multi-reaction mode (MRM). More details on this method and recovery rates can be found in the supporting information (SI, Tables SI-1, SI-2, and SI-3). The recovery rates of the used isotopic-labeled internal standards were evaluated and summarized in the SI (Table SI-4). All PFAS analyses were performed in duplicate.

### Leaching tests

Two types of laboratory leaching tests were conducted using doubly demineralized water (generated by a Milli-Q® device) as eluent.

1. Column percolation tests

Column percolation tests were conducted in duplicate in accordance with DIN 19528 (2023) using an automated column test device (ecoTech GmbH, Bonn, Germany). Usually, DIN 19528 requires sampling at 0.3, 1.0, 2.0, and 4.0 l/kg. A longer test duration up to an L/S around 10 l/kg was chosen to enable comparison with batch tests at L/S of 10 l/kg. Five fractions at approximately 0.3, 1.0, 2.0, 5.0, and 10.0 l/kg were taken and immediately preserved at 4 °C before analysis. In contrast to the higher flow rate for the saturation phase (2 h contact time) stipulated by DIN 19528 at the time of the experiments, a contact time of 5 h was used for both the saturation and the percolation phase, i.e., the flow rate was kept constant throughout both phases of the experiment, which is between what is stipulated in the old and in the updated version of the standard. The flow rate of individual columns may vary slightly, especially for different samples, due to differences in the packing of the columns. The soils were introduced to the columns in layers and gently compacted using a special rammer as required by the standard. Test conditions are shown in Table 2. Further information on the calculation of contact time and flow rate based on porosity, bulk density, and information on the calculation of release is given in the SI. The test conditions of the German leaching tests are very similar to the tests standardized on the European and international levels (Krüger et al. 2012; DIN 19529 2023), so the results of this study are transferable.

**Table 2:** Test conditions of the column leaching test for all soil samples (RA1, RA2, TE, TX)

	Unit	RA1	RA2	TE	TX
Material of column	-	Glass	Glass	Glass	Glass
Material of tubing^a^	-	FEP	FEP	FEP	FEP
Column diameter	cm	5.86	5.86	5.86	5.86
Filling height	cm	30	30	30	30
Sample mass	g	706	949	1058	1045
Particle density	g/cm^3^	2.38	2.54	2.66	2.58
Bulk density	g/cm^3^	0.83	1.17	1.30	1.29
Porosity	%	65	54	51	50
1-pore volume	ml	526	436	414	404
Flow rate	ml/min	1.76	1.50	1.37	1.37
Contact time sample/eluent	h	5.00	4.85	5.03	4.91

^a^FEP (a co-polymer of hexafluoropropylene and tetrafluoroethylene) was identified in a previous study (Kalbe et al. 2014) as the most suitable material for the tubing, due to its very smooth surface

2. Batch tests

Batch tests were performed in duplicate and at two different liquid-to-solid ratios (L/S 2 l/kg and 10 l/kg) in accordance with DIN 19529 (2023) by shaking the sample for 24 h end-over-end at 7 rpm. The two L/S ratios were applied because there are different legal requirements in the different areas of mineral waste characterization (L/S 10 l/kg for landfill and L/S 2 l/kg for reuse classification) in Germany (Kalbe et al. 2008; Lopez Meza et al. 2010). The subsequent separation of the liquid and solid phases is susceptible to influence by sorption of the analytes. Therefore, three separation steps were compared (see Table 3). A stainless-steel pressure filtration device was used for membrane and glass fiber filtration. The centrifugation vessels were made of stainless steel, as well.

**Table 3.**
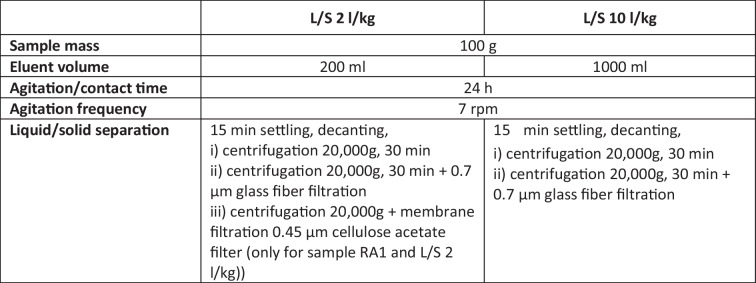
Test conditions of batch tests for two different liquid/solid ratios (L/S)

### Eluate analysis

All eluates were divided into five aliquots for subsequent analysis comprising the determination of four accompanying parameters as well as the PFCA and PFSA analysis. Conductivity (DIN EN 27888 1993), pH value (DIN EN ISO 10523 2012), and turbidity (DIN EN ISO 7027 2016) were measured immediately after sampling. Filters of 0.45 µm cut-off and a TOC VCPH (Shimadzu, Duisburg, Germany) were used to determine DOC (dissolved organic carbon) concentrations in the eluates after filtration, in accordance with DIN EN 1484 (2019). We calculated DOC by subtracting DIC (dissolved inorganic carbon) from DC (dissolved carbon).

Every parameter was measured in duplicate. The fifth aliquot was used for PFAS analysis of eluates. We briefly describe the PFAS analysis of eluates above and in greater detail in the SI.

### Quality assurance

The eluent applied for the leaching tests and eluates of blank tests using the two leaching procedures were evaluated for cross contamination from the materials of the test devices. In the case of column tests, the same amount of filter sand used was included in the blank test as in the tests with the sample materials. In the batch tests, the blank tests included the filtration of the eluate using the same equipment and filter materials. Glass bottles were used for the leaching tests as they fit best to the devices: the shaker for the batch tests and the connections of the collection bottles to the automated column test system. This is not optimal for the investigation of PFAS due to possible sorption losses. The eluates were therefore immediately transferred into polypropylene bottles for the analyses directly after liquid/solid separation in batch tests and immediately after completion of the collection of a fraction in column tests. We did not use PTFE lids for glass bottles used for batch tests or for collecting column test eluates. The caps used to close the columns were made of PVC.

All PFAS measured in the eluent and in the eluates of the blank tests were below the limit of quantification (LOQ). To determine possible matrix effects, the extracts were diluted with methanol by a factor of 10, enabling the recognition of effects such as ion suppression. These possible matrix effects cannot occur during the analysis of the eluates, since in accordance with the DIN standard used, solid-phase extraction is used to purify and enrich the analytes. The recovery rates of the isotopic-labeled internal PFAS standard are similar in both analytical methods and can be found in the SI together with the LOQ and LOD values for the soil samples and the eluates.

## Results and discussion

### Soil characteristics and total contents of PFAS

Table 4 shows the basic characteristics of the four soil samples: the main differences can be found for pH value, the content of organic carbon (measured as loss on ignition) and the total carbon content (TC) and the amount of carbonate.

**Table 4:** Basic characteristics of all soil samples investigated (RA1, RA2, TE, TX) including standard deviation (number of independent replicates in parenthesis)

	RA1	RA2	TE	TX
Residual moisture content (%)	4.66 ± 0.08 (3)	1.10 ± 0.13 (3)	0.70 ± 0.46 (3)	1.36 ± 0.45 (3)
pH (-)	7.62 ± 0.08 (2)	7.74 ± 0.06 (2)	8.49 ± 0.25 (2)	6.41 ± 0.12 (2)
Conductivity (µS/cm)	112 ± 0.1 (2)	160 ± 0.1 (2)	116 ± 15 (2)	40 ± 0.1 (2)
Carbonate content (%)	0.31 ± 0.10 (3)	1.06 ± 0.07 (3)	2.69 ± 0.06 (3)	0.37 ± 0.06 (3)
Loss on ignition (%) Total carbon content (TC) (%) Soil type	12.14 ± 0.78 (2) 5.74 (1) medium sand	4.67 ± 0.07 (2) 2.23 (1) coarse sand	0.58 ± 0.08 (2) 0.45 (1) medium sand	3.19 ± 0.01 (2) 1.82(1) fine sand

The individual PFAS substances found in the four soils (see content in solid matter in Tables 5, 6, 7, and 8, standard deviations can be found in Table SI-5) are typical of their contamination sources, as reported also in other papers (Guelfo and Higgins 2013; Høisæter et al. 2019; Kotthoff et al. 2020; Rayner et al. 2022; Bierbaum et al. 2023) which give an overview of typical contamination levels also. For eluates, a solid-phase extraction is applied in accordance with the DIN standard (DIN 38407–42 2011), which is a cleanup and preconcentration step in a single procedure. The mass of the sample taken for the leaching tests (100 to 900 g) is greater than for determining the PFAS content analysis (0.5 g). So, the measurement of PFAS concentration in eluates is more sensitive regarding the LOQ. Therefore, it is possible that the amounts of substances are below the LOQ in the methanol extracts of solid matter, whereas the same substance was detected in the eluate (this applies to PFPeA in RA2, TE, TX, and to PFHpA in all soils (partly not for L/S 10 l/kg) and PFDA in TX.

**Table 5:** Content of the PFAS substances measured in solid matter in sample RA1 compared with the released amount and percentage of released amount by the leaching tests at liquid-to-solid ratios L/S 2 and 10 l/kg (all analyses at least in duplicate)

	RA1	RA1	RA1	RA1	RA1	RA1	RA1	RA1	RA1
	Content in solid matter	Eluate: cumulative release at L/S 2 l/kg column test	Released amount up to L/S 2 l/kg column test	Eluate: cumulative release at L/S 10 l/kg column test	Released amount up to L/S 10 l/kg column test	Released amount up to L/S 2 l/kg batch test	Released amount up to L/S 2 l/kg batch test	Released amount up to L/S 10 l/kg batch test	Released amount up to L/S 10 l/kg batch test
	µg/kg dm	µg/kg	%	µg/kg	%	µg/kg	%	µg/kg	%
PFBA	10.25	0.21	2.04	0.36	3.51	0.93	9.08	2.90	28.28
PFPeA	29.10	0.79	2.71	1.23	4.24	2.89	9.92	9.35	32.13
PFHxA	28.07	0.26	0.92	0.55	1.96	0.53	1.88	1.54	5.50
PFHpA	27.87	2.32	8.33	3.25	11.67	3.04	10.92	8.86	31.80
PFOA	83.45	7.20	8.63	9.26	11.10	4.53	5.43	15.87	19.01
PFNA	40.18	2.22	5.52	4.94	12.29	1.79	4.45	4.60	11.45
PFDA	400.43	4.76	1.19	35.07	8.76	3.26	0.81	24.15	6.03
PFBS	3.75	0.01	0.29	0.02	0.42	0.02	0.42	0.06	1.60
PFHxS	1.28	0.16	12.23	0.22	17.16	0.14	11.25	0.24	18.94
PFOS	640.52	17.03	2.66	87.94	13.73	11.96	1.87	64.37	10.05
Sum	1264.91	34.95		142.85		29.08		131.94	
		*n* = 2		*n* = 2		*n* = 3		*n* = 3

**Table 6:** Content of the PFAS substances measured in solid matter in sample RA2 compared with the released amount and percentage of released amount by the leaching tests at liquid-to-solid ratios L/S 2 and 10 l/kg (all analyses at least in duplicate)

	RA2	RA2	RA2	RA2	RA2	RA2	RA2	RA2	RA2
	Content in solid matter	Eluate: cumulative release at L/S 2 l/kg column test	Released amount up to L/S 2 l/kg column test	Eluate: cumulative release at L/S 10 l/kg column test	Released amount up to L/S 10 l/kg column test	Released amount up to L/S 2 l/kg batch test	Released amount up to L/S 2 l/kg batch test	Released amount up to L/S 10 l/kg batch test	Released amount up to L/S 10 l/kg batch test
	µg/kg dm	µg/kg	%	µg/kg	%	µg/kg	%	µg/kg	%
PFBA	2.26	0.19	8.52	0.31	13.92	0.76	33.72	1.94	85.90
PFPeA	< 1.11	2.27	-	4.17	-	9.97	-	18.76	-
PFHxA	6.57	0.13	2.03	0.37	5.61	0.34	5.23	0.61	9.36
PFHpA	1.09	0.92	84.12	1.83	167.35	2.12	193.54	5.16	471.55
PFOA	25.81	3.53	13.66	4.65	18.01	5.88	22.76	9.83	38.09
PFNA	20.24	3.32	16.40	9.09	44.92	2.41	11.92	10.34	51.08
PFDA	205.37	6.11	2.97	34.02	16.56	6.09	2.97	23.47	11.43
PFBS	3.40	0.004	0.12	0.007	0.20	< 0.0012	< 0.04	0.04	1.12
PFHxS	1.06	0.003	0.29	0.003	0.29	0.0004	0.04	0.02	2.05
PFOS	1.85	0.50	26.95	2.47	133.42	0.32	17.36	1.70	91.62
Sum	267.66	16.98		56.93		27.90		71.87	
		*n* = 2		*n* = 2		*n* = 3		*n* = 3

**Table 7:** Content of the PFAS substances measured in solid matter in sample TE compared with the released amount and percentage of released amount by the leaching tests at liquid-to-solid ratios L/S 2 and 10 l/kg (all analyses at least in duplicate)

	TE	TE	TE	TE	TE	TE	TE	TE	TE
	Content in solid matter	Eluate: cumulative release at L/S 2 l/kg column test	Released amount up to L/S 2 l/kg column test	Eluate: cumulative release at L/S 10 l/kg column test	Released amount up to L/S 10 l/kg column test	Released amount up to L/S 2 l/kg batch test	Released amount up to L/S 2 l/kg batch test	Released amount up to L/S 10 l/kg batch test	Released amount up to L/S 10 l/kg batch test
	µg/kg dm	µg/kg	%	µg/kg	%	µg/kg	%	µg/kg	%
PFBA	2.80	0.01	0.33	0.01	0.33	0.27	9.68	2.22	79.26
PFPeA	< 1.11	0.06	-	0.06	-	0.36	-	1.24	-
PFHxA	20.56	0.08	0.40	0.12	0.57	0.37	1.81	1.53	7.43
PFHpA	< 0.41	0.002	-	0.002	-	0.01	-	0.03	-
PFOA	4.81	0.29	6.13	0.40	8.36	0.45	9.46	1.72	35.79
PFNA	< 0.22	< 0.0017	-	< 0.0085	-	< 0.0017	-	< 0.0085	-
PFDA	< 0.43	< 0.0015	-	< 0.0075	-	< 0.0015	-	< 0.0075	-
PFBS	5.48	0.43	7.77	0.44	8.07	1.62	29.56	3.03	55.23
PFHxS	46.87	6.05	12.90	7.25	15.47	12.35	26.36	29.43	62.79
PFOS	971.49	41.46	4.27	93.29	9.60	36.97	3.81	119.01	12.25
Sum	1052.01	48.38		101.57		52.41		158.20	
		*n* = 2		*n* = 2		*n* = 3		*n* = 3

**Table 8:** Content of the PFAS substances measured in solid matter in sample TX compared with the released amount and percentage of released amount by the leaching tests at liquid-to-solid ratios L/S 2 and 10 l/kg (all analyses at least in duplicate)

	TX	TX	TX	TX	TX	TX	TX	TX	TX
	Content in solid matter	Eluate: cumulative release at L/S 2 l/kg column test	Released amount up to L/S 2 l/kg column test	Eluate: cumulative release at L/S 10 l/kg column test	Released amount up to L/S 10 l/kg column test	Released amount up to L/S 2 l/kg batch test	Released amount up to L/S 2 l/kg batch test	Released amount up to L/S 10 l/kg batch test	Released amount up to L/S 10 l/kg batch test
	µg/kg dm	µg/kg	%	µg/kg	%	µg/kg	%	µg/kg	%
PFBA	0.93	0.03	2.95	0.03	2.95	0.15	15.81	< 0.012	-
PFPeA	< 1.11	0.01	-	0.01	-	0.07	-	< 0.006	-
PFHxA	3.53	0.03	0.72	0.03	0.95	0.04	1.25	0.13	3.70
PFHpA	< 0.41	0.01	-	0.01	-	0.003	-	< 0.004	-
PFOA	2.00	0.14	6.80	0.14	6.80	0.11	5.57	0.42	21.05
PFNA	< 0.22	< 0.0017	-	< 0.0085	-	< 0.0017	-	< 0.0085	-
PFDA	< 0.43	0.02	-	0.03	-	0.003	-	< 0.0075	-
PFBS	3.49	0.12	3.30	0.13	3.65	0.10	2.84	0.32	9.03
PFHxS	4.84	2.60	53.73	5.26	108.70	3.12	64.40	10.81	223.33
PFOS	177.93	20.80	11.69	52.61	29.57	16.97	9.54	64.71	36.37
Sum	192.73	23.74		58.24		20.56		76.38	
		*n* = 2		*n* = 2		*n* = 3		*n* = 3	

The total PFAS content is determined via one-step extraction using methanol as the extraction agent, which is probably less efficient than using water (Hale et al. 2017). In three samples, PFOS was present in the highest total content of the 10 measured PFCA and PFSA: 641 µg/kg in RA1, 971 µg/kg in TE, and 178 µg/kg in TX). There were also high levels of PFDA in RA1 (400 µg/kg), while all other compounds had levels between 1.3 and 83 µg/kg (Table 5). In RA2, PFDA was the compound with the highest content (205 µg/kg), followed by the other substances with contents between 1.1 and 26 µg/kg, except PFPeA, whose level was below the LOQ (Table 6). In the TE sample, more PFHxS was present (47 µg/kg) than the other substances (5.5 to 20.6 µg/kg, Table 7). In the TX sample, only five other PFCA and PFSA were detected with levels between 0.93 and 4.8 µg/kg (Table 8).

### Comparison of the leaching behavior of the PFAS species investigated

**Fig. 1:**
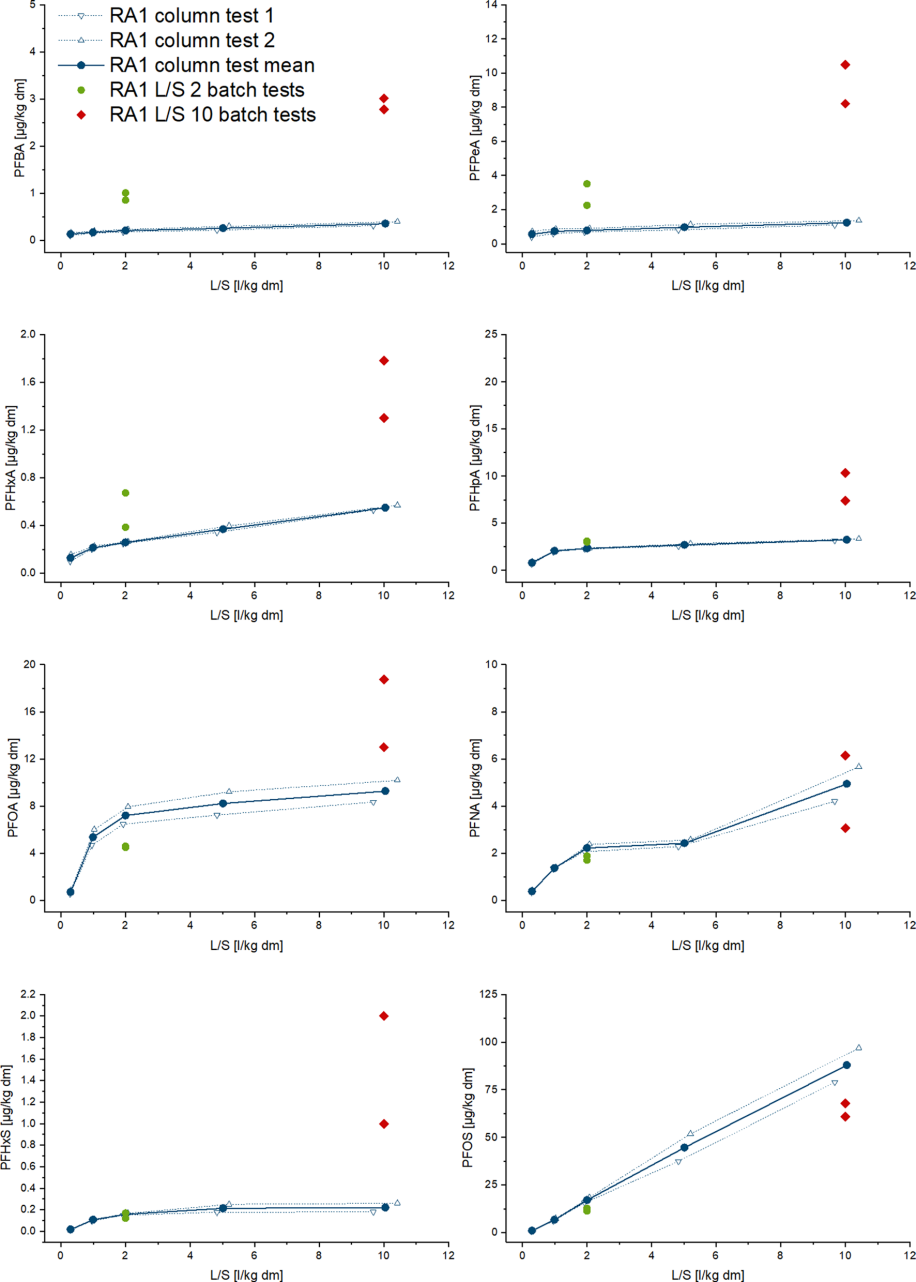
Cumulative release of PFAS in eluates from column and batch tests depending on liquid/solid ratio (L/S) in sample RA1 (selected substances with measurable concentrations)

**Fig. 2:**
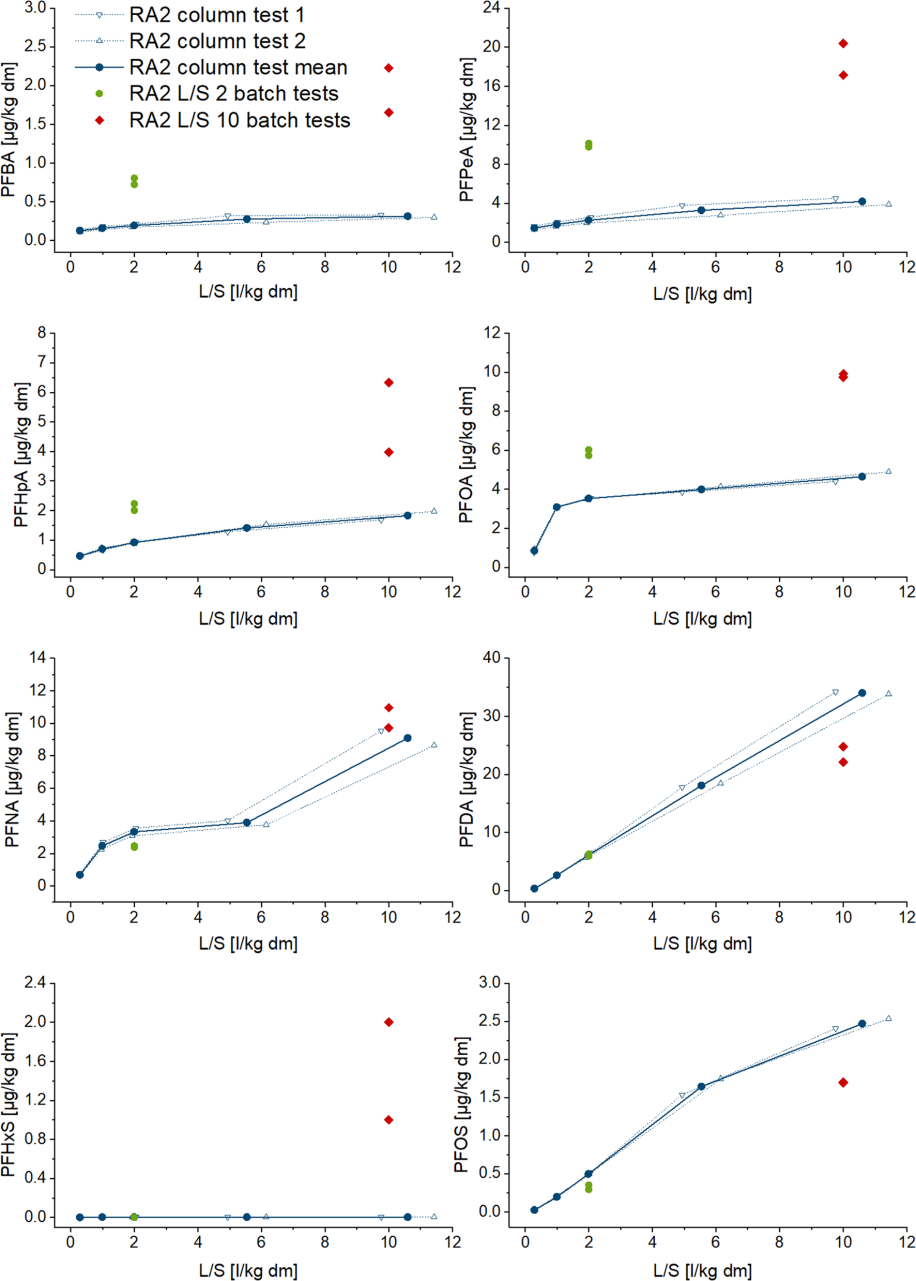
Cumulative release of PFAS in eluates from column and batch tests depending on liquid/solid ratio (L/S) in sample RA2 (selected substances with measurable concentrations)

**Fig. 3:**
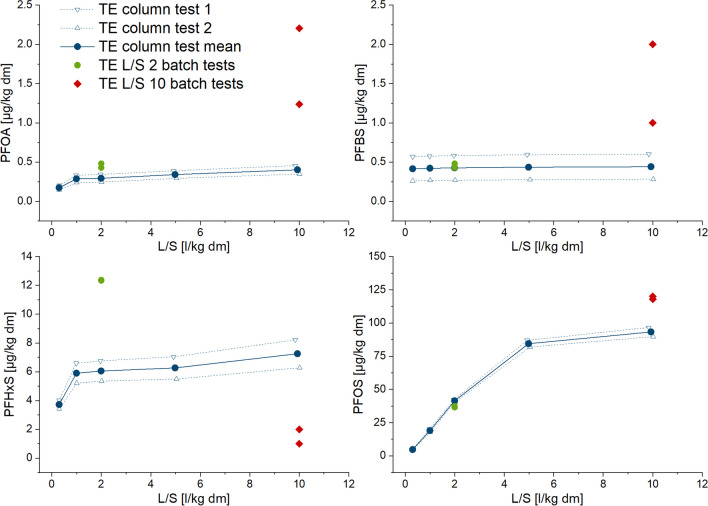
Cumulative release of PFAS in eluates from column and batch tests depending on liquid/solid ratio (L/S) in sample TE (selected substances with measurable concentrations)

**Fig. 4:**
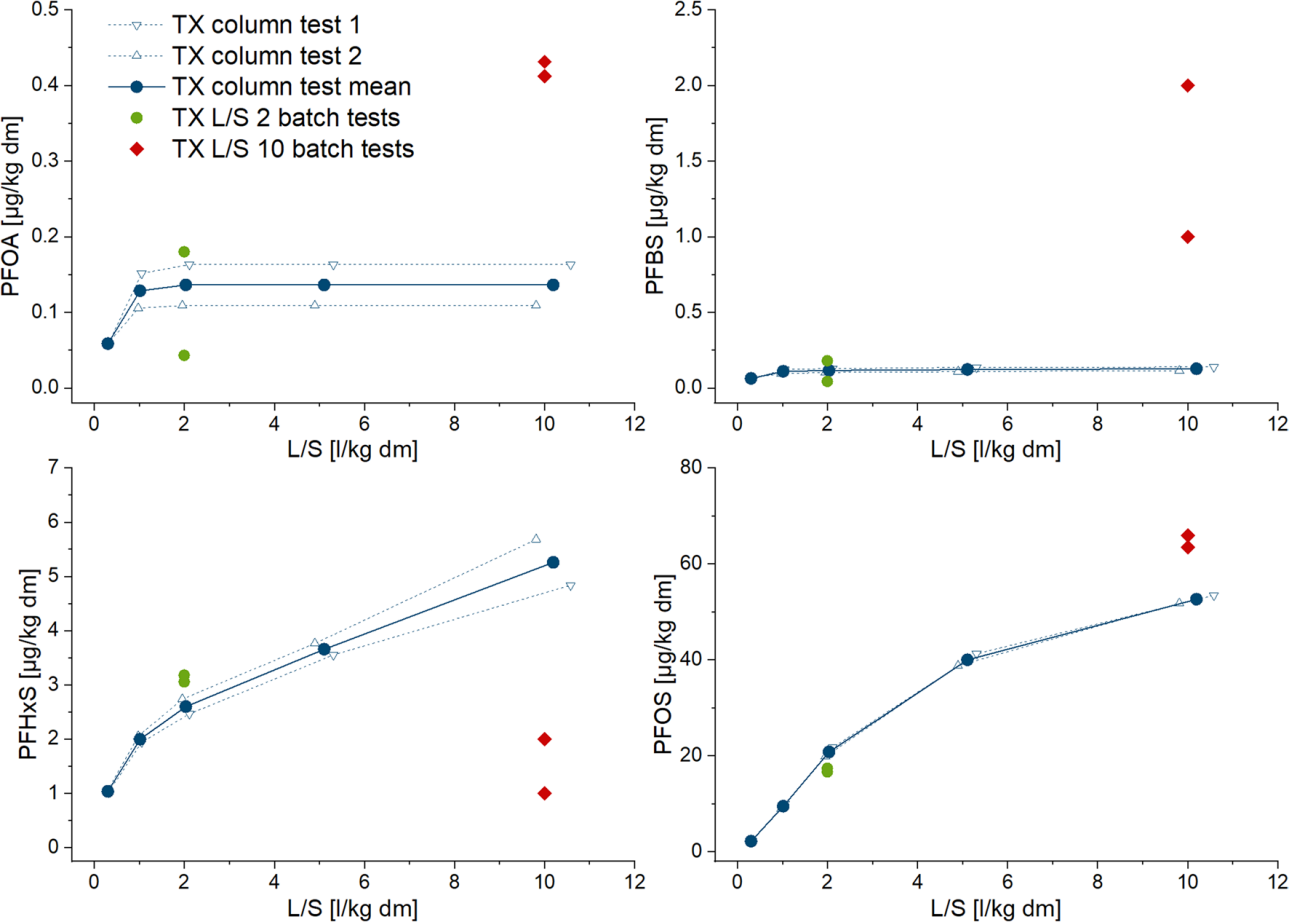
Cumulative release of PFAS in eluates from column and batch tests depending on liquid/solid ratio (L/S) in sample TX (selected substances with measurable concentrations)

To be able to compare the results of the two types of leaching tests, the cumulative release of selected PFAS in the column and batch tests is shown in Figs. 1, 2, 3, and 4. Graphs showing the associated concentrations for column tests and the accompanying parameters pH, turbidity, and TOC for column and batch tests are provided in the SI (Figure SI-1 to SI-8). Additionally, for comparative purposes, Tables 5, 6, 7, and 8 contain the cumulative release up to L/S 2 and 10 l/kg obtained from batch and column tests, as well as the respective release rate of the selected PFAS related to their total contents in all soils investigated. This is the only way that the results from column and batch tests can be compared. Concentrations are not directly comparable, because in batch tests, the eluate is collected for one specified L/S and in column tests for certain L/S ranges. The graphs showing the concentrations measured are provided in the SI (Figures SI-1 to SI-4) to illustrate orders of magnitude.

Depletion of some PFAS seems to occur, as there is a plateau in the release curves (Figs. 1, 2, 3, and 4), but it can be assumed that after the first wash-off (first flush), low solubilities then slow down the release, leading to eluate concentrations below LOQ (refer also to Figure SI-1 to 4 in SI). Non-equilibrium conditions and a diffusion-controlled release or even retarded intraparticular diffusion are probably the prevailing conditions with increasing L/S, as shown especially for organic substances (Grathwohl and Susset 2009; Finkel and Grathwohl 2017). In general, the different sources are reflected by the results, and the two samples with a similar contamination source each (paper sludge and fire extinguishing foam) show similar distribution patterns of PFAS release.

The RA samples contained more measurable PFAS in the eluates than were found in the eluates of the TE/TX samples (Tables 5, 6, 7, and 8). Release curves were calculated for eight of the substances considered: PFBA, PFPeA, PFHpA, PFOA, PFNA, PFHxS, and PFOS for both samples, while PFHxA and PFDA could be calculated only for RA1 and RA2 respectively (Figs. 1 and 2). The curves show that for PFOS, PFNA, PFHXa (RA1) as well as PFDA (RA2), the release will continue up to a higher liquid-to-solid ratio. In the RA2 sample, PFPeA was below the detection limit for solid matter analysis. Nevertheless, a low release in the column tests was observed, due to the greater sensitivity of eluate analysis. The percentage of release of individual compounds varies between the two RA samples without a clear pattern (Tables 5 and 6). In general, the percentage released is higher for the RA2 samples: for RA1, the margin for individual compounds varies between 0.42 and 17% and for the RA2 sample between 0.2 and 167%. The release rates higher than 100% of PFHpA and PFOS could be caused by the uncertainty of the measurement in solid matter. Overall, 11% and 21% of the sum of the considered PFAS in solid matter were released, up to an L/S of 10 l/kg in the column tests using the RA1 and RA2 samples, respectively. The smaller release of the RA1 sample might be linked to higher TOC content (Sepulvado et al. 2011; Hale et al. 2017; Kabiri et al. 2021; Röhler et al. 2021).

For the TE and TX samples, only PFOS, PFHxS, PFBS, and PFOA were detected in more than one fraction of the column percolation test, making it possible to calculate cumulative release curves (Figs. 3 and 4). There was no detection of PFHxA and PFNA in the eluate samples. The only two substances with increasing cumulative release up to an L/S of 10 l/kg were PFOS and PFHxS. The two other compounds show a rapid decline in eluate concentrations with increasing L/S and are frequently detectable only in the first eluate fraction. PFOS and PFHxS showed the greatest measured cumulative release of the PFAS, amounting to 93 µg/kg and 7.3 µg/kg respectively at an L/S of 10 l/kg for the TE sample as well as 29 µg/kg and 109 µg/kg for the TX sample (Tables 7 and 8). Comparison with the solid matter content (Tables 7 and 8) reveals that 9.6% (TE) and 30% (TX) of the total content of the sum of the PFAS considered were released up to L/S 10 l/kg in the column test. Høisæter et al. (2019) reported results from unsaturated column tests using a contaminated soil taken at an airport and irrigated at infiltration rates of 4.9 and 9.7 mm/day. A release rate of 0.006% and 0.05% respectively was observed for PFOS (which had a share of 96% of the total content in the soil) at the lower and higher infiltration rate, whereas 100% and 87% respectively were found for PFBS, 47% for PFHxS, and around 6% for the sum of 23 PFAS for both infiltration rates. The column percolation tests in this study were performed at much higher flow velocities (41 cm/day for TE and 54 cm/day for RA) for a shorter time and under saturated conditions. Hale et al. (Hale et al. 2017) reported high solid matter contents of PFOS (up to 2400 µg/kg) in samples from a firefighting training facility and concentrations ranging up to 157 µg/l in batch tests at an L/S of 10 l/kg. The released amount of PFOS compared with the total content was calculated beginning at 54% and partly over 100%, which might be due to the presence of precursor substances and their transformation products (Bierbaum et al. 2023; Weidemann et al. 2022).

Bolan et al. (2021) provide a good overview of references to contamination levels of soil and soil leachates. The soils used in our study were contaminated with PFAS several years before sampling. In the case of the TE sample, the airport and therefore the training site for firefighting were closed about 10 years before sampling. In the case of the RA sample, the compost contaminated with PFAS during agricultural management was used in a period around 12 to 8 years before sampling, when mobile PFAS had already been released (Röhler et al. 2021). Furthermore, it is known that the sorption of organic compounds in soil can change due to aging processes (Naidu and Bolan 2008; An et al. 2017; Schaefer et al. 2021). Organic compounds adsorbed by solid matrices such as soils and sediments can be divided into fractions with different availabilities and showing different desorption behavior: the easily available fraction is desorbed within days, while this process may take years for the less-available fraction (Ortega-Calvo et al. 2015). Nguyen et al. (2022) revealed the faster release of short-chained PFAS (*C* ≤ 6) from soils contaminated with AFFF. Due to the long period of field aging and exposure to rainwater, it can be assumed that the easily available fraction in our samples had already been leached in the field. A subsequent column percolation experiment then shows overall lower releases.

To describe the leaching behavior of PFAS from soil, complex processes driven by the physical and chemical properties of both the substances and the soils (Li et al. 2018) must be considered. The sorption of PFAS to soil depends not only on the content of organic matter (Milinovic et al. 2015; Bierbaum et al. 2023), but among other factors also on the organic matter’s charge and pH value (Campos Pereira et al. 2018; Nguyen et al. 2020; Rayner et al. 2022). For example, partitioning between soil and porewater depends on chain length (Nguyen et al. 2020). For compounds with the same chain length, the head group leads to differences: perfluoroalkane sulfonates (PFSA) are found to bind more strongly to soils and especially to humic substances than perfluoroalkyl carboxylates (PFCA) and are less or more slowly released (Campos Pereira et al. 2018). For the RA1 sample with the highest content on organic matter measured by loss on ignition and a soil pH of 7.62 (Table 4), this can be confirmed with both leaching test types, e.g., by comparing PFOA and PFOS (Table 5). PFOS is released at lower rates than PFOA. This trend was not observed in sample RA2 with lower organic matter content (Table 4).

In our study, the release rates vary between < 1% and > 100%. No trend depending on the chain length was observed. However, as discussed above, this might be caused by the time that passed between soil contamination and sampling.

Leaching tests are common tools to determine the potential release of contaminants from soil. However, there are limitations in predicting the actual release of PFAS, since the hydrological conditions in the field cannot be precisely simulated by laboratory leaching tests (Schaefer et al. 2023), and equilibrium conditions therefore differ (Guelfo et al. 2020; Nguyen et al. 2022).

### Comparison of batch and column leaching test results

The two types of leaching tests were compared to evaluate the agreement of results. Static equilibrium is given in a batch test, but dynamic equilibrium prevails in column tests. Additionally, the contact time between the soil and the eluent, the type of contact (shaking or percolation), and the mechanical impact on the sample differ between the two test types (Tables 2 and 3).

Graphs showing the concentrations for column tests are found in SI (Figures SI-1 to 4), and concentration data for batch tests are listed in SI (Table SI-6).

Figures 1 and 2 show the cumulative release of selected PFAS comparatively for batch and column tests. They show that column test results are somewhat more reproducible than those of batch tests, i.e., in our study, the replicates of column tests vary less. The fact that the sample mass used in the tests was six to nine times greater in column tests than in batch tests and weaker influences from preparing the column test eluates for analysis are very likely the reasons for this observation (Table 2 and Table 3). This was also found during the validation of the German leaching standards, especially for organic substances (DIN 19528 2023; DIN 19529 2023).

Comparing the release of PFAS using both leaching test types (Tables 5, 6, 7, and 8) reveals that batch tests mostly overestimate the release of shorter-chain PFAS, whereby the effect was greater with carboxylic than with sulfonic acids. Considering the percentage of release, this effect was less pronounced in the RA1 sample, possibly due to the greater content of organic matter (Table 5). The higher release in batch tests is assumed to be related to the agitation step (shaking) and therefore a higher mobilization of colloids, as well as the possible friction between particles.

The concentrations of some substances in the TX sample were not measurable in the batch test eluates at L/S 10 l/kg only which seems to be merely a dilution effect resulting in concentrations below LOQ (Figure SI-4, Table 8). The first column test fractions may contain higher concentrations due to surface wash-off and the low L/S ratios, leading to analytical detectability greater than in the batch test at L/S 10 l/kg (one fraction in one operation).

**Fig. 5:**
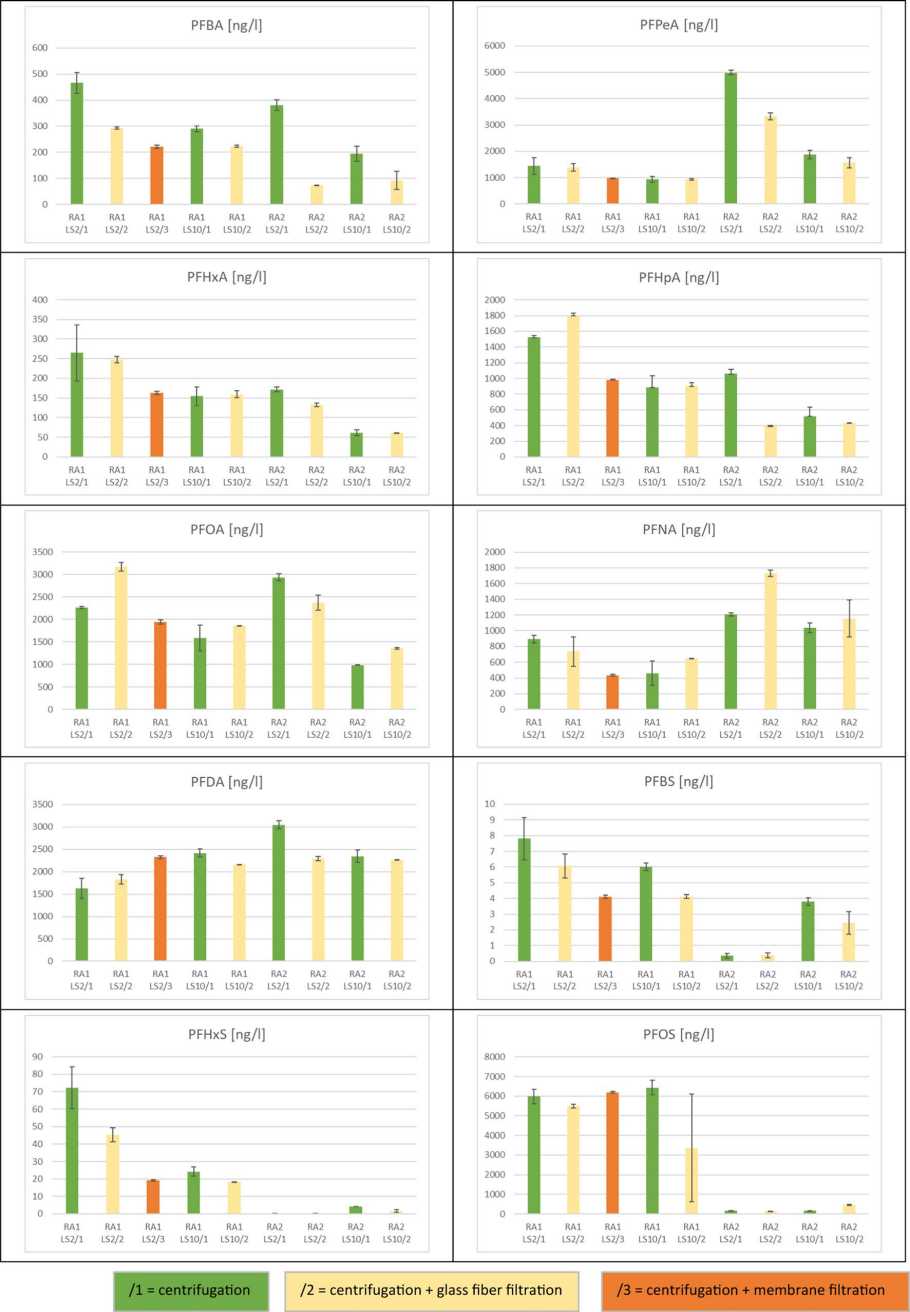
Effect of eluate pretreatment on PFAS concentration in the eluates before analysis for the soil samples RA1 and RA2 from agriculturally used areas near Rastatt (Germany)

**Fig. 6:**
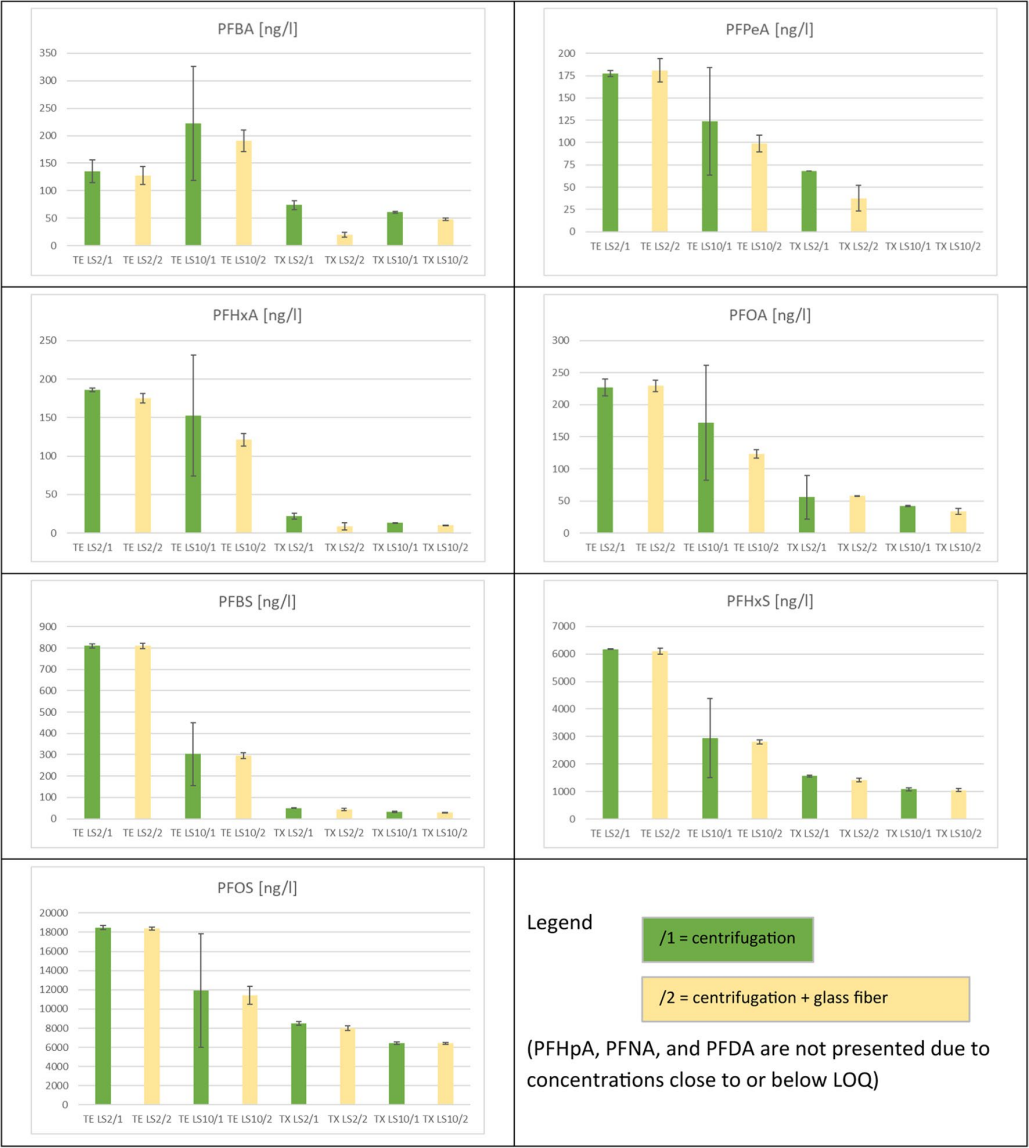
Effect of eluate pretreatment on PFAS concentration in the eluates before analysis for the soil samples TE and TX from former airports in Berlin (Tempelhof and Tegel)

The application of mechanical energy during shaking might induce higher eluate concentrations in batch-leaching tests. Moreover, differences between the results of the two test types may result from somewhat different conditions for equilibrium establishment and different contact time (see Tables 2 Table 3). However, this has not yet been tested comparatively for the two test types. In this study, all samples were dried at ambient air temperature to support sample preparation and homogenization. Differences between column and batch test can therefore not be caused by the preparation of the soil samples. However, differences between the two types of tests in eluate preparation before analysis may influence the comparability of the leaching test results. The scope of the standard applied for PFAS analysis (DIN 38407–42 2011) addresses water with a very low amount of colloidal particles, such as drinking water and groundwater. Eluates obtained from soil usually contain more colloidal particles, especially when the organic matter content of the soil is greater and must be pretreated (filtered or centrifuged) before analysis (Krüger et al. 2012). It has been shown, e.g., for polycyclic aromatic hydrocarbons as an important and well-examined substance group of organic contaminants, that membrane filtration is not as appropriate as glass fiber filtration, due to sorption of the analytes on the filter material (DIN 19529 2023). However, this should be tested for every substance group and filter type. Therefore, in this study, batch test eluates were subdivided and three pretreatment steps were applied: (i) centrifugation (standard condition), (ii) centrifugation followed by a glass fiber filtration (approximately 0.7 µm cut-off) as optionally proposed in DIN 19529, and (iii) centrifugation followed by membrane filtration using cellulose acetate filters of 0.45 µm cut-off only for batch test eluates at L/S 2 l/kg of the sample with the highest content in organic matter, RA1 (Figs. 5 and 6 and Table SI-6). Eluates from the column experiments were directly analyzed for PFAS without centrifugation or filtration, since the turbidity of the eluates was always below 100 FNU, which is the criterion of the applied procedure (DIN 19528 2023).

The turbidities of the batch test eluates were greater than those of the column test eluates except for the TE sample, which had the lowest organic matter content (Figure SI-5 to 8). Moreover, the variability of turbidity in the batch tests was mostly greater than the variability in the column tests, very likely due to the additional preparation step for the batch test eluates. Differences resulting from the additional glass fiber filtration of batch test eluates were mostly not obvious for both L/S ratios applied (Figs. 5 and 6 and Table SI-6). When membrane filtration is applied (only for RA1, L/S 2 l/kg), the results indicate some retention compared with glass fiber filtration for almost all PFAS. However, this was observed only in one sample material. Sörengård et al. (2020) had tested the sorption effect of 0.45-µm cellulose acetate membrane filters, among other filter types, with 21 different PFAS substances. The sorption losses on the membrane filters accounted for up to 21% and were somewhat lower in the presence of DOC compared with pure water, whereby sorption was greater for longer-chained perfluoroalkyl sulfonates than for perfluoroalkyl carboxylates. Glass fiber filters were not included in this study.

Our study’s comparison of centrifugation and glass fiber filtration did not reveal a clear pattern. There is only a slight tendency to more retention of PFAS in samples RA1 and RA2 when glass fiber filtration is used, which was observed mainly with PFBA, PFBS, and PFHxS. This effect is more pronounced for PFBA in sample RA2, probably due to eluates with less DOC (Figure SI-6) than in sample RA1 (Figure SI-5), which is in line with the findings of Sörengård et al. (2020). The DOC dependence can also be confirmed by comparing this effect for L/S 2 and 10 eluates.

There is no clear difference between the eluate concentrations in the TE and TX samples after centrifugation and glass fiber filtration, especially for the sulfonic acids. Additionally, no dependence on chain length or on the L/S ratio was observed in these samples. A statistical evaluation of the data on eluate preparation before analysis was not carried out, as the data set is not sufficient for this.

To be on the safe side regarding losses of analytes, we recommend avoiding membrane filtration and waiving additional glass fiber filtration where possible, since PFAS are known to stick especially to glassware. Any additional contact to the surfaces of equipment may lead to losses of analytes (Lath et al. 2019).

It has to be noted that we cannot exclude the influence of degradation of precursors during the tests which may hamper the comparisons of column and batch tests in this study. Especially, PFHpA, PFOA, or PFNA are known as typical degradation products of the precursors 6:2 diPAPs, 8:2 diPAPs, and diPAPs with longer perfluorinated chains which are reported to be present in considerable amounts in the soils of the Rastatt area which is the origin of the soils RA1 and RA2 (Bierbaum et al. 2023; Weidemann et al. 2022). The amount of degradation products generally increases with reaction time, so we would expect this degradation to be higher in the column percolation tests. As shown in Figs. 1, 2, 3, and 4, the release of PFCAs is generally higher in the batch tests than in the column tests, suggesting that a strong influence of degradation on our results is unlikely to have occurred.

## Conclusions

This study focused on comparing two types of laboratory leaching tests in order to evaluate whether the results can be considered equivalent for risk assessment purposes. Column percolation tests are more laborious than batch tests but reveal a more realistic picture and offer a time-dependent observation of substance release. Another advantage of column tests over batch tests is the greater mass of the sample taken for one test, making it more representative of the sample taken in the field and leading to a better repeatability. A further reason for this observation could be that column test eluates usually require no eluate treatment before analysis, while the necessary filtration for batch test eluates is prone to errors that need to be carefully assessed, especially considering that DIN 38407–42 (2011) does not allow any filtration for analysis. The results show that any unnecessary step of sample preparation of the eluates should be avoided if possible. The possible omission of the liquid/solid separation of the eluates obtained from column tests is a clear advantage over batch leaching tests. However, losses of PFAS cannot be completely avoided, due to the multiple potential contacts of the eluates with the surfaces of the equipment used in both test types.

Our test indicates that batch test results overestimate the release of PFAS from soil. This conclusion should be verified by testing more soils with varying characteristics and PFAS distribution patterns. The examination of more substances including precursors could help to interpret mass balances.

Although the soil samples investigated represent different soil types, conclusions about the influence of soil characteristics (e.g., the content of organic matter and sesquioxides, pH value, residual moisture, and PFAS substance distribution pattern) on leaching behavior have yet to be clarified, since they cannot be derived from only four soil samples. This requires more systematic investigation.

It has become apparent that the lower sensitivity of the standardized analytical procedure for PFAS in solid matter compared with the procedure for the eluates is a disadvantage for such investigations. In part, the released amount of some individual PFAS could not be calculated, since the amount of PFAS in the soils was below the detection limit. To overcome these limitations in the final evaluation of the results, the insufficient limits of detection and of quantification in the analysis of PFAS in solid matter need to be improved in the German standard. Here, a better separation of matrix components, especially humic substances from the target PFAS, must be optimized to avoid ion suppression.

Due to the increasing number of contamination cases involving PFAS worldwide, the responsible standardization committee needs to immediately consider how to improve leaching test procedures and analytical methods. In current standards, especially for ground and surface water, the portfolio of PFAS analytes of interest is increased from 10 to 30 (e.g., ISO 21675 2019). This analyte spectrum should be considered in further studies.

## Supplementary Information

Below is the link to the electronic supplementary material.

Supplementary file1 (DOCX 735 KB)

## Data Availability

The authors declare that the data supporting the findings of this study are available within the paper and its Supplementary Information file.

## Abbreviations

*DOC*: Dissolved organic carbon
*FEP*: Perfluorethylenpropylene
*TC*: Total carbon content
*K*_d_: Distribution coefficient in soil
*K*_oc_: Organic carbon normalized adsorption coefficient
*K*_ow_: Octanol-water partition coefficient
*LC**–**MS/MS*: Liquid chromatography mass spectrometry
*LOQ*: Limit of quantification
*L/S*: Liquid-to-solid ratio
*SPE*: Solid phase extraction
*dm*: Dry matter
*rpm*: Revolutions per minute
PFSA: Perfluorosulfonic acids
PFCA: Perfluorocarboxylic acids
PFBA: Perfluoro-*n*-butanoic acid
PFPeA: Perfluoro-*n*-pentanoic acid
PFHxA: Perfluoro-*n*-hexanoic acid
PFHpA: Perfluoro-*n*-heptanoic acid
PFOA: Perfluoro-*n*-octanoic acid
PFNA: Perfluoro-*n*-nonanoic acid
PFDA: Perfluoro-*n*-dodecanoic acid
PFBS: Perfluoro butane sulfonic acid
PFHxS: Perfluoro hexane sulfonic acid
PFOS: Perfluorooctanoic sulfonic acid
PTFE: Polytetrafluoroethylene

## References

[CR1] Ahrens L, Bundschuh M (2014) Fate and effects of poly- and perfluoroalkyl substances in the aquatic environment: a review. Environ Toxicol Chem 33(9):1921–1929. 10.1002/etc.2663 24924660 10.1002/etc.2663

[CR2] An X, Xiao B, Di X, Dong H, Tang H (2017) Research progress on aging of organic pollutants in geosorbents: a review. Acta Geochimica 36(1):27–43. 10.1007/s11631-016-0129-z

[CR3] Anderson RH, Long GC, Porter RC, Anderson JK (2016) Occurrence of select perfluoroalkyl substances at U.S. Air Force aqueous film-forming foam release sites other than fire-training areas: field-validation of critical fate and transport properties. Chemosphere 150:678–685. 10.1016/j.chemosphere.2016.01.014 26786021 10.1016/j.chemosphere.2016.01.014

[CR4] Arcadis U.S. Inc. (2020) Review of models for evaluating per- and polyfluoroalkyl substances in land applied residuals and biosolids – an assessment of fate and transport models for groundwater leaching, surface water runoff, and plant uptake (V1.1). In: National Council for Air and Stream Improvement, Inc., Cary, NC, USA

[CR5] Baduel C, Paxman CJ, Mueller JF (2015) Perfluoroalkyl substances in a firefighting training ground (FTG), distribution and potential future release. J Hazard Mater 296:46–53. 10.1016/j.jhazmat.2015.03.007 25966923 10.1016/j.jhazmat.2015.03.007

[CR6] Banzhaf S, Hebig KH (2016) Use of column experiments to investigate the fate of organic micropollutants – a review. Hydrol Earth Syst Sci 20(9):3719–3737. 10.5194/hess-20-3719-2016

[CR7] Bierbaum T, Klaas N, Braun J, Nürenberg G, Lange FT, Haslauer C (2023) Immobilization of per- and polyfluoroalkyl substances (PFAS): comparison of leaching behavior by three different leaching tests. Sci Total Environ 876:162588. 10.1016/j.scitotenv.2023.162588 36871732 10.1016/j.scitotenv.2023.162588

[CR8] Blaine AC, Rich CD, Hundal LS, Lau C, Mills MA, Harris KM, Higgins CP (2013) Uptake of perfluoroalkyl acids into edible crops via land applied biosolids: field and greenhouse studies. Environ Sci Technol 47(24):14062–14069. 10.1021/es403094q 24206563 10.1021/es403094q

[CR9] Bolan N, Sarkar B, Yan Y, Li Q, Wijesekara H, Kannan K, Tsang DCW, Schauerte M, Bosch J, Noll H, Ok YS, Scheckel K, Kumpiene J, Gobindlal K, Kah M, Sperry J, Kirkham MB, Wang H, Tsang YF, Hou D, Rinklebe J (2021) Remediation of poly- and perfluoroalkyl substances (PFAS) contaminated soils – to mobilize or to immobilize or to degrade? J Hazard Mater 401:123892. 10.1016/j.jhazmat.2020.123892 33113753 10.1016/j.jhazmat.2020.123892 PMC8025151

[CR10] Brunn H, Arnold G, Körner W, Rippen G, Steinhäuser KG, Valentin I (2023) PFAS: forever chemicals – persistent, bioaccumulative and mobile. Reviewing the status and the need for their phase out and remediation of contaminated sites. Environ Sci Eur 35(1):20. 10.1186/s12302-023-00721-8

[CR11] Butt CM, Muir DCG, Mabury SA (2014) Biotransformation pathways of fluorotelomer-based polyfluoroalkyl substances: a review. Environ Toxicol Chem 33(2):243–267. 10.1002/etc.2407 24114778 10.1002/etc.2407

[CR12] Campos Pereira H, Ullberg M, Kleja DB, Gustafsson JP, Ahrens L (2018) Sorption of perfluoroalkyl substances (PFASs) to an organic soil horizon – effect of cation composition and pH. Chemosphere 207:183–191. 10.1016/j.chemosphere.2018.05.012 29793030 10.1016/j.chemosphere.2018.05.012

[CR13] Chen S, Zhou Y, Meng J, Wang T (2018) Seasonal and annual variations in removal efficiency of perfluoroalkyl substances by different wastewater treatment processes. Environ Pollut 242:2059–2067. 10.1016/j.envpol.2018.06.078 30231460 10.1016/j.envpol.2018.06.078

[CR14] Coggan TL, Moodie D, Kolobaric A, Szabo D, Shimeta J, Crosbie ND, Lee E, Fernandes M, Clarke BO (2019) An investigation into per- and polyfluoroalkyl substances (PFAS) in nineteen Australian wastewater treatment plants (WWTPs). Heliyon 5(8):e02316. 10.1016/j.heliyon.2019.e02316 31485522 10.1016/j.heliyon.2019.e02316 PMC6716228

[CR15] DIN 18128:2002–12 (n.d.) Baugrund – Untersuchung von Bodenproben – Bestimmung des Glühverlustes (Soil – investigation and testing – determination of ignition loss), German Institute for Standardization

[CR16] DIN 19528:2023–07 (n.d.) Elution von Feststoffen – Perkolationsverfahren zur gemeinsamen Untersuchung des Elutionsverhaltens von anorganischen und organischen Stoffen (Leaching of solid materials – percolation method for the joint examination of the leaching behaviour of inorganic and organic substances), German Institute for Standardization

[CR17] DIN 19529:2023–07 (n.d.) Elution von Feststoffen – Schüttelverfahren zur Untersuchung des Elutionsverhaltens von anorganischen und organischen Stoffen bei einem Wasser/Feststoff-Verhältnis von 2 l/kg (Leaching of solid materials – batch test for the examination of the leaching behaviour of inorganic and organic substances at a liquid to solid ratio of 2 l/kg), German Institute for Standardization

[CR18] DIN 38407–42:2011–03 (n.d.) Deutsche Einheitsverfahren zur Wasser-, Abwasser- und Schlammuntersuchung – Gemeinsam erfassbare Stoffgruppen (Gruppe F) – Teil 42: Bestimmung ausgewählter polyfluorierter Verbindungen (PFC) in Wasser – Verfahren mittels Hochleistungs-Flüssigkeitschromatographie und massenspektrometrischer Detektion (HPLC-MS/MS) nach Fest-Flüssig-Extraktion (F 42) (German standard methods for the examination of water, waste water, and sludge – jointly determinable substances (group F) – Part 42: determination of selected polyfluorinated compounds (PFC) in water – method using high performance liquid chromatography and mass spectrometric detection (HPLC/MS-MS) after solid-liquid extraction (F 42)), German Institute for Standardization

[CR19] DIN 38414–14:2011–08 (n.d.) Deutsches Einheitsverfahren zur Wasser, Abwasser- und Schlammuntesuchung – Schlamm und Sedimente (Gruppe S) – Teil 14: Bestimmung ausgewählter polyfluorierter Verbindungen (PFC) in Schlamm, Kompost, und Boden – Verfahren mittels Hochleistungs-Flüssigkeitschromatographie und massenspektrometrischer Detektion (HPLC-MS/MS) (S 14) (German standard methods for the examination of water, waste water, and sludge – sludge and sediments (group S) – Part 14: determination of selected polyfluorinated compounds (PFC) in sludge, compost, and soil – method using high performance liquid chromatography and mass spectrometric detection (HPLC-MS/MS) (S 14)), German Institute for Standardization

[CR20] DIN 66137–2:2019–03 (n.d.) Bestimmung der Dichte fester Stoffe – Teil 2: Gaspyknometrie (Determination of solid state density – part 2: gaspycnometry), German Institute for Standardization

[CR21] DIN EN 1484:2019–04 (n.d.) Water analysis – guidelines for the determination of total organic carbon (TOC) and dissolved organic carbon (DOC), German Institute for Standardization

[CR22] DIN EN 27888:1993–11 (n.d.) Water quality; determination of electrical conductivity (ISO 7888:1985), German Institute for Standardization

[CR23] DIN EN ISO 10523:2012–04 (n.d.) Water quality – determination of pH (ISO 10523:2008), German Institute for Standardization

[CR24] DIN EN ISO 10693:2014–06 (n.d.) Soil quality – determination of carbonate content – volumetric method, German Institute for Standardization

[CR25] DIN EN ISO 7027:2016–11 (n.d.) Water quality – determination of turbidity – part 1: quantitative methods (ISO 7027–1:2016), German Institute for Standardization

[CR26] DIN ISO 11465:1996–12 (n.d.) Soil quality; determination of dry matter and water content on a mass basis; gravimetric method, German Institute for Standardization

[CR27] ECHA (2023) Annex XV restriction report – proposal for a restriction: Per- and polyfluoroalkyl substances (PFASs), 22.03.2023, https://echa.europa.eu/documents/10162/f605d4b5-7c17-7414-8823-b49b9fd43aea. Accessed 2023-12-01

[CR28] EN 12457–2:2002–09 (n.d.) Characterization of waste – leaching; compliance test for leaching of granular and sludges – part 2: one stage batch test at a liquid to solid ratio of 10 l/kg with particle size below 4 mm (without or with size reduction)

[CR29] EN 12457–4:2002–09 (n.d.) Characterization of waste – leaching; compliance test for leaching of granular waste materials and sludges – part 4: one stage batch test at a liquid to solid ratio of 10 l/kg for materials with particle size below 10 mm (without or with limited size reduction)

[CR30] EN 14405:2017–03 (n.d.) Characterization of waste – leaching behaviour tests – up-flow percolation test (under specified conditions), European Committee for Standardization

[CR31] US EPA (2019a) Leaching environmental assessment framework (LEAF) how-to guide. Environmental Protection Agency, U.S. https://www.epa.gov/sites/default/files/2019-05/documents/final_leaching_environmental_assessment_framework_leaf_how-to_guide.pdf

[CR32] European Commission (2020) Regulation (EU) 2020/784 of the European Parliament and of the Council of 8 April 2020 on persistent organic pollutants amending Annex I to Regulation (EU) 2019/1021 as regards the listing of perfluorooctanoic acid (PFOA), its salts and PFOA-related compounds. https://eur-lex.europa.eu/legal-content/EN/TXT/?uri=CELEX:32020R0784

[CR33] Fiedler H, Kallenborn R, de Boer J, Sydnes LK (2019) The stockholm convention: a tool for the global regulation of persistent organic pollutants. Chem Int 41(2):4–11. 10.1515/ci-2019-0202

[CR34] Finkel M, Grathwohl P (2017) Impact of pre-equilibration and diffusion limited release kinetics on effluent concentration in column leaching tests: insights from numerical simulations. Waste Manage 63:58–73. 10.1016/j.wasman.2016.11.031 10.1016/j.wasman.2016.11.031 27919562

[CR35] Garrabrants AC, Kosson DS, R. D, Kariher P, Seignette PFAB, van der Sloot HA, Stefanski L, Baldwin M (2012) Interlaboratory validation of the leaching environmental assessment framework (LEAF) method 1314 and method 1315. US Environmental Protection Agency EPA-600/R-12/624. https://nepis.epa.gov/Exe/ZyPURL.cgi?Dockey=P100FAFC.TXT

[CR36] Grathwohl P (2014) On equilibration of pore water in column leaching tests. Waste Manage 34(5):908–918. 10.1016/j.wasman.2014.02.012 10.1016/j.wasman.2014.02.012 24636008

[CR37] Grathwohl P, Susset B (2009) Comparison of percolation to batch and sequential leaching tests: theory and data. Waste Manage 29(10):2681–2688. 10.1016/j.wasman.2009.05.016 10.1016/j.wasman.2009.05.016 19576753

[CR38] Guelfo JL, Higgins CP (2013) Subsurface transport potential of perfluoroalkyl acids at aqueous film-forming foam (AFFF)-impacted sites. Environ Sci Technol 47(9):4164–4171. 10.1021/es3048043 23566120 10.1021/es3048043

[CR39] Guelfo JL, Wunsch A, McCray J, Stults JF, Higgins CP (2020) Subsurface transport potential of perfluoroalkyl acids (PFAAs): column experiments and modeling. J Contam Hydrol 233:103661. 10.1016/j.jconhyd.2020.103661 32535327 10.1016/j.jconhyd.2020.103661

[CR40] Guo B, Zeng J, Brusseau ML (2020) A mathematical model for the release, transport, and retention of per- and polyfluoroalkyl substances (PFAS) in the vadose zone. Water Resour Res 56(2):e2019WR026667. 10.1029/2019WR026667 10.1029/2019wr026667 PMC7673302 33223573

[CR41] Hale SE, Arp HPH, Slinde GA, Wade EJ, Bjørseth K, Breedveld GD, Straith BF, Moe KG, Jartun M, Høisæter Å (2017) Sorbent amendment as a remediation strategy to reduce PFAS mobility and leaching in a contaminated sandy soil from a Norwegian firefighting training facility. Chemosphere 171:9–18. 10.1016/j.chemosphere.2016.12.057 28002769 10.1016/j.chemosphere.2016.12.057

[CR42] Høisæter Å, Pfaff A, Breedveld GD (2019) Leaching and transport of PFAS from aqueous film-forming foam (AFFF) in the unsaturated soil at a firefighting training facility under cold climatic conditions. J Contam Hydrol 222:112–122. 10.1016/j.jconhyd.2019.02.010 30878240 10.1016/j.jconhyd.2019.02.010

[CR43] ISO 21268–1:2019 (n.d.) Soil quality – Leaching procedures for subsequent chemical and ecotoxicological testing of soil and soil-like materials – part 1: batch test using a liquid to solid ratio of 2 l/kg dry matter

[CR44] ISO 21268–2:2019 (n.d.) Soil quality – leaching procedures for subsequent chemical and ecotoxicological testing of soil and soil-like material – part 2: batch test using a liquid to solid ratio of 10 l/kg dry matter

[CR45] ISO 21268–3:2019 (n.d.) Soil quality – leaching procedures for subsequent chemical and ecotoxicological testing of soil and soil-like materials – part 3: up-flow percolation test

[CR46] DIN ISO 10390:2005–12 (n.d.) Soil quality – determination of pH, German Institute for Standardization

[CR47] ITRC (2020) PFAS Fact Sheets – physical and chemical properties table for select PFAS (Excel file, updated April 2020). https://pfas-1.itrcweb.org/fact-sheets/

[CR48] Kabiri S, McLaughlin MJ (2021) Durability of sorption of per- and polyfluorinated alkyl substances in soils immobilized using common adsorbents: 2. Effects of repeated leaching, temperature extremes, ionic strength and competing ions. Sci Total Environ 766, 144718. 10.1016/j.scitotenv.2020.144718 10.1016/j.scitotenv.2020.144718 33536123

[CR49] Kalbe U, Berger W, Eckardt J, Simon FG (2008) Evaluation of leaching and extraction procedures for soil and waste. Waste Manage 28(6):1027–1038. 10.1016/j.wasman.2007.03.008 10.1016/j.wasman.2007.03.008 17531462

[CR50] Kalbe U, Bandow N, Bredow A, Mathies H, Piechotta C (2014) Column leaching tests on soils containing less investigated organic pollutants. J Geochem Exploration 147(Part B):291–297. 10.1016/j.gexplo.2014.06.014

[CR51] Kotthoff M, Fliedner A, Rüdel H, Göckener B, Bücking M, Biegel-Engler A, Koschorreck J (2020) Per- and polyfluoroalkyl substances in the German environment – levels and patterns in different matrices. Sci Total Environ 740:140116. 10.1016/j.scitotenv.2020.140116 32559548 10.1016/j.scitotenv.2020.140116

[CR52] Krüger O, Kalbe U, Berger W, Simon FG, López Meza S (2012) Leaching experiments on the release of heavy metals and PAH from soil and waste materials. J Hazard Mater 207–208:51–55. 10.1016/j.jhazmat.2011.02.016 10.1016/j.jhazmat.2011.02.016 21377270

[CR53] Lange FT, Scheurer M, Bierreth C, Borho W, Seeger A-K, Dreher P, Nöltner T (2020) Air-drying of soil samples – a crucial step in the determination of leachable concentrations of per- and polyfluoroalkyl substances. Chemosphere 128745. 10.1016/j.chemosphere.2020.128745 10.1016/j.chemosphere.2020.128745 33131729

[CR54] Lath S, Knight ER, Navarro DA, Kookana RS, McLaughlin MJ (2019) Sorption of PFOA onto different laboratory materials: filter membranes and centrifuge tubes. Chemosphere 222:671–678. 10.1016/j.chemosphere.2019.01.096 30735967 10.1016/j.chemosphere.2019.01.096

[CR55] Li Y, Oliver DP, Kookana RS (2018) A critical analysis of published data to discern the role of soil and sediment properties in determining sorption of per and polyfluoroalkyl substances (PFASs). Sci Total Environ 628–629:110–120. 10.1016/j.scitotenv.2018.01.167 10.1016/j.scitotenv.2018.01.167 29428853

[CR56] Link GW, Reeves DM, Cassidy DP, Coffin ES (2024) Per- and polyfluoroalkyl substances (PFAS) in final treated solids (Biosolids) from 190 Michigan wastewater treatment plants. J Hazard Mater 463:132734. 10.1016/j.jhazmat.2023.132734 37922581 10.1016/j.jhazmat.2023.132734

[CR57] López Meza S, Garrabrants AC, van der Sloot H, Kosson DS (2008) Comparison of the release of constituents from granular materials under batch and column testing. Waste Manage 28(10):1853–1867 10.1016/j.wasman.2007.11.009 18242972

[CR58] Lopez Meza S, Kalbe U, Berger W, Simon F-G (2010) Effect of contact time on the release of contaminants from granular waste materials during column leaching experiments. Waste Manage 30(4):565–571 10.1016/j.wasman.2009.11.022 20034777

[CR59] McLachlan MS, Felizeter S, Klein M, Kotthoff M, De Voogt P (2019) Fate of a perfluoroalkyl acid mixture in an agricultural soil studied in lysimeters. Chemosphere 223:180–187. 10.1016/j.chemosphere.2019.02.012 30776763 10.1016/j.chemosphere.2019.02.012

[CR60] Meegoda JN, Kewalramani JA, Li B, Marsh RW (2020) A review of the applications, environmental release, and remediation technologies of per- and polyfluoroalkyl substances. Int J Environ Res Public Health 17(21). 10.3390/ijerph17218117 10.3390/ijerph17218117 PMC7663283 33153160

[CR61] Milinovic J, Lacorte S, Vidal M, Rigol A (2015) Sorption behaviour of perfluoroalkyl substances in soils. Sci Total Environ 511:63–71. 10.1016/j.scitotenv.2014.12.017 25531590 10.1016/j.scitotenv.2014.12.017

[CR62] Naidu R, Bolan NS (2008) Chapter 2 Contaminant chemistry in soils: key concepts and bioavailability. In: Hartemink AE, McBratney AB, Naidu R (eds) Developments in Soil Science, vol 32. Elsevier, pp 9–37

[CR63] Naka A, Yasutaka T, Sakanakura H, Kalbe U, Watanabe Y, Inoba S, Takeo M, Inui T, Katsumi T, Fujikawa T, Sato K, Higashino K, Someya M (2016) Column percolation test for contaminated soils: key factors for standardization. J Hazard Mater 320:326–340. 10.1016/j.jhazmat.2016.08.046 27565857 10.1016/j.jhazmat.2016.08.046

[CR64] Nguyen TMH, Bräunig J, Thompson K, Thompson J, Kabiri S, Navarro DA, Kookana RS, Grimison C, Barnes CM, Higgins CP, McLaughlin MJ, Mueller JF (2020) Influences of chemical properties, soil properties, and solution pH on soil-water partitioning coefficients of per- and polyfluoroalkyl substances (PFASs). Environ Sci Technol 54(24):15883–15892. 10.1021/acs.est.0c05705 33249833 10.1021/acs.est.0c05705

[CR65] Nguyen TMH, Bräunig J, Kookana RS, Kaserzon SL, Knight ER, Vo HNP, Kabiri S, Navarro DA, Grimison C, Riddell N, Higgins CP, McLaughlin MJ, Mueller JF (2022) Assessment of mobilization potential of per- and polyfluoroalkyl substances for soil remediation. Environ Sci Technol 56(14):10030–10041. 10.1021/acs.est.2c00401 35763608 10.1021/acs.est.2c00401

[CR66] Ortega-Calvo J-J, Harmsen J, Parsons JR, Semple KT, Aitken MD, Ajao C, Eadsforth C, Galay-Burgos M, Naidu R, Oliver R, Peijnenburg WJGM, Römbke J, Streck G, Versonnen B (2015) From bioavailability science to regulation of organic chemicals. Environ Sci Technol 49(17):10255–10264. 10.1021/acs.est.5b02412 26230485 10.1021/acs.est.5b02412

[CR67] Posner S (2012) Perfluorinated compounds: occurrence and cases in products, in: Knepper TP and Lange FT (eds.), Polyfluorinated chemicals and transformation products, 25–39, Berlin, Heidelberg: Springer Berlin Heidelberg

[CR68] Rayner JL, Slee D, Falvey S, Kookana R, Bekele E, Stevenson G, Lee A, Davis GB (2022) Laboratory batch representation of PFAS leaching from aged field soils: intercomparison across new and standard approaches. Sci Total Environ 838:156562. 10.1016/j.scitotenv.2022.156562 35690200 10.1016/j.scitotenv.2022.156562

[CR69] Röhler K, Haluska AA, Susset B, Liu B, Grathwohl P (2021) Long-term behavior of PFAS in contaminated agricultural soils in Germany. J Contam Hydrol 241:103812. 10.1016/j.jconhyd.2021.103812 34245996 10.1016/j.jconhyd.2021.103812

[CR70] Schaefer CE, Nguyen D, Christie E, Shea S, Higgins CP, Field JA (2021) Desorption of poly- and perfluoroalkyl substances from soil historically impacted with aqueous film-forming foam. J Environ Eng 147(2):06020006. 10.1061/(ASCE)EE.1943-7870.0001846

[CR71] Schaefer CE, Lavorgna GM, Lippincott DR, Nguyen D, Schaum A, Higgins CP, Field J (2023) Leaching of perfluoroalkyl acids during unsaturated zone flushing at a field site impacted with aqueous film forming foam. Environ Sci Technol 57(5):1940–1948. 10.1021/acs.est.2c06903 36689630 10.1021/acs.est.2c06903

[CR72] Sepulvado JG, Blaine AC, Hundal LS, Higgins CP (2011) Occurrence and fate of perfluorochemicals in soil following the land application of municipal biosolids. Environ Sci Technol 45(19):8106–8112. 10.1021/es103903d 21446724 10.1021/es103903d

[CR73] Sörengård M, Franke V, Tröger R, Ahrens L (2020) Losses of poly- and perfluoroalkyl substances to syringe filter materials. J Chromatography A 1609. 10.1016/j.chroma.2019.460430 10.1016/j.chroma.2019.460430 31445806

[CR74] Stahl T, Riebe RA, Falk S, Failing K, Brunn H (2013) Long-term lysimeter experiment to investigate the leaching of perfluoroalkyl substances (PFASs) and the carry-over from soil to plants: results of a pilot study. J Agric Food Chem 61(8):1784–1793. 10.1021/jf305003h 23379692 10.1021/jf305003h

[CR75] United Nations Environment Progamme (2004) 15th Stockholm Convention on persistent organic pollutants, Chapter XXVII Environment. https://treaties.un.org/pages/ViewDetails.aspx?src=TREATY&mtdsg_no=XXVII-15&chapter=27

[CR76] US EPA (2019b) Estimation programs interface suite™ for Microsoft® windows, v 4.11. United States environmental protection agency, Washington, DC, USA. https://www.epa.gov/tsca-screening-tools/download-episuitetm-estimation-program-interface-v411

[CR77] van der Veen AMH, Nater DAG (1993) Third international rolduc symposium on coal science and technology and related processes, sample preparation from bulk samples: an overview. Fuel Process Technol 36(1):1–7. 10.1016/0378-3820(93)90003-M

[CR78] Van Glubt S, Brusseau ML, Yan N, Huang D, Khan N, Carroll KC (2021) Column versus batch methods for measuring PFOS and PFOA sorption to geomedia. Environ Pollut 268:115917. 10.1016/j.envpol.2020.115917 33143983 10.1016/j.envpol.2020.115917 PMC7746577

[CR79] Wang Z, Xie Z, Mi W, Möller A, Wolschke H, Ebinghaus R (2015) Neutral poly/per-fluoroalkyl substances in air from the atlantic to the southern ocean and in Antarctic snow. Environ Sci Technol 49(13):7770–7775. 10.1021/acs.est.5b00920 26052844 10.1021/acs.est.5b00920

[CR80] Washington JW, Rankin K, Libelo EL, Lynch DG, Cyterski M (2019) Determining global background soil PFAS loads and the fluorotelomer-based polymer degradation rates that can account for these loads. Sci Total Environ 651:2444–2449. 10.1016/j.scitotenv.2018.10.071 30336434 10.1016/j.scitotenv.2018.10.071 PMC7957346

[CR81] Weidemann E, Lämmer R, Stahl T, Göckener B, Bücking M, Breuer J, Kowalczyk J, Just H, Boeddinghaus RS, Gassmann M (2022) Leaching and transformation of perfluoroalkyl acids and polyfluoroalkyl phosphate diesters in unsaturated soil column studies. Environ Toxicol Chem 41(9):2065–2077 35751449 10.1002/etc.5417

[CR82] Zareitalabad P, Siemens J, Hamer M, Amelung W (2013) Perfluorooctanoic acid (PFOA) and perfluorooctanesulfonic acid (PFOS) in surface waters, sediments, soils and wastewater – a review on concentrations and distribution coefficients. Chemosphere 91(6):725–732. 10.1016/j.chemosphere.2013.02.024 23498059 10.1016/j.chemosphere.2013.02.024

