# Supplementary material for: Comparing PFAS analysis in batch leaching and column leaching tests
Source: Environ Sci Pollut Res Int. 2024 Nov 22;31(57):65233–51. doi: 10.1007/s11356-024-35510-0 (PMC11631822; doi:10.1007/s11356-024-35510-0)
Supplement: Supplementary file 1 — Supplementary file1 (DOCX 735 KB) [file 11356_2024_35510_MOESM1_ESM.docx]

**Leaching of PFAS – Comparison of laboratory leaching tests**

Ute Kalbe^a^, Christian Piechotta^a^, Nicole Bandow^a,b^

^a^ Federal Institute for Materials Research and Testing – BAM, 12200 Berlin, Germany

^b^ current address: German Environment Agency, Corrensplatz 1, 14195 Berlin

**Detailed description of PFAS analysis**

**Analysis of soil samples [1]**

0.5 g soil sample was extracted with 2.5 ml methanol in an ultra-sonic bath for 30 min after addition of 50 µl of ^13^C-labelled internal standard solution. After settling for 1h, an aliquot of 500 µl supernatant was filtered using a 0.45 µm cellulose filter and diluted with 500 µl ultra-pure water (MiliQ). Analyses for soil samples were performed using an Agilent 1260 HPLC and an AB SCIEX TSQ 6500 as mass selective detector. 7 µl of the samples was injected and separated on a Nucleodur C 18 Pyramid pre-column (8 mm x 3 mm, 3 µm) and a Nucleodur C18 Pyramid column (125 mm x 2mm; 3 µm) (both Macherey- Nagel, Düren, Germany) at 35 °C and a flow rate of 0.3 ml/min using the following gradient program using water with 10 mMol ammonium acetate (eluent A) and methanol (eluent B): the eluent composition of 75 % A and 25 % B at the beginning changed in 9 min to 25 % A and 75 % B. The ion source was operated at 425 °C and with an ion spray voltage of-1200 V and all measurements were executed in the multireaction mode (MRM).

**Analysis of eluates [2]**

An aliquot of the unfiltered sample of 40 ml is taken from a Flacon tube. The samples are then spiked with a mix of isotope-labelled internal standards with the required target concentrations. For this purpose, the calculated amount of internal standard is added to the sample and is controlled gravimetrically. The sample vessel is swayed vigorously in the closed state for homogenization. The sample is then processed with the following steps (Table SI-1) using a weak anion exchanger (here Chromabond PFAS) and an SPE vacuum chamber. The volumes of the individual SPE solutions (elution solution, wash solution, etc.) are added by using the multiple dispenser (25 ml attachment). The sample volume to be analyzed is added to the SPE cartridge with the help of an attached reservoir column. 15 ml Falcon tubes are used to collect the elutates. All steps are usually carried out at atmospheric pressure. For particle containing samples, a slight vacuum (negative pressure) may be used. For direct measurement of the SPE eluate, an aliquot of 500 μl is taken and mixed with 500 μl of water. This solution can then be analyzed directly by LC-MS/MS. If a concentration step is necessary, the SPE eluates can be concentrated to dryness within 45 min in the Falcon tubes in a concentration unit (e.g., TurboVap LV at 40 °C and 10 psi nitrogen flow). The residue is then reconstituted using a suitable volume (e.g., 200 μl) of the solvent mixture of water/methanol 50%/50% (v/v) using a test tube shaker. The resulting solution can then be transferred to an LC vial and analyzed by LC-MS/MS.

**Table SI-1: SPE parameter**

| **Step** | **Solvent / volume** |
| --- | --- |
| Conditioning I | 4 ml elution solution |
| Conditioning II | 4 ml methanol |
| Conditioning III | 4 ml water |
| Load sample | 40 ml sample |
| Washing step | 4 ml washing solution* |
| Rinsing of the sample vessel; collecting of the eluate in a Falcon tube |  |
| Elution | 4 ml elution solution** |

* Washing solution for SPE: 25 mM ammonium acetate buffer (pH 4.0).

** Elution solution for SPE: methanol with ammonia containing 0.1% by volume.

**Table SI-2: Qualifier and quantifier ions in the multi-reaction mode MS method part**

| PFC species |  | Molecular ion in Da | 1. Product ion in Da | 2. Product ion in Da | DP in Volt | CE in Volt |
| --- | --- | --- | --- | --- | --- | --- |
| Heptafluorobutyric acid | PFBA | 213.0 | 168.8 | - | - 45 | - 17 |
| Nonafluoropentaonic acid | PFPeA | 263.0 | 219.0 | - | - 45 | - 17 |
| Undecahexanoic acid | PFHxA | 313.0 | 269.0 | 119.0 | - 45 | - 17 |
| Dodecafluoroheptanoic acid | PFHpA | 363.0 | 319.0 | 169.0 | - 45 | - 17 |
| Pentadecafluorooctanoic acid | PFOA | 413.0 | 369.0 | 169.0 | - 50 | - 17 |
| Heptadecafluorononanoic acid | PFNA | 463.0 | 419.0 | 219.0 | - 50 | - 17 |
| Nonadecafluorodecanoic acid | PFDA | 513.0 | 469.0 | 219.0 | - 50 | - 17 |
| Perfluorobutanesulfonic acid | PFBS | 299.0 | 80.0 | 99.0 | - 65 | - 52 |
| Tridecafluorohexane-1-sulfonic acid | PFHxS | 399.0 | 80.0 | 99.0 | - 85 | - 70 |
| Heptadecafluoro-1-octanesulfonic acid | PFOS | 499.0 | 80.0 | 99.0 | - 105 | - 85 |

**Table SI-3: Isotopically labelled internal standards used for the quantification of PFAS of interest**

| Target PFAS species |  | Internal standards |  |
| --- | --- | --- | --- |
| Heptafluorobutyric acid | PFBA | [13C4] Heptafluorobutyric acid | PFBA_INT |
| Nonafluoropentaonic acid | PFPeA | [1,2-13C2] Undecahexanoic acid | PFHxA_INT |
| Undecahexanoic acid | PFHxA | [1,2-13C2] Undecahexanoic acid | PFHxA_INT |
| Dodecafluoroheptanoic acid | PFHpA | [1, 2, 3, 4-13C4] Pentadecafluorooctanoic acid | PFOA_INT |
| Pentadecafluorooctanoic acid | PFOA | [1, 2, 3, 4-13C4] Pentadecafluorooctanoic acid | PFOA_INT |
| Heptadecafluorononanoic acid | PFNA | [1, 2, 3, 4-13C4] Pentadecafluorooctanoic acid | PFOA_INT |
| Nonadecafluorodecanoic acid | PFDA | [1, 2, 3, 4-13C4] Pentadecafluorooctanoic acid | PFOA_INT |
| Perfluorobutanesulfonic acid | PFBS | [1, 2, 3, 4-13C4] Heptadecafluoro-1-octanesulfonic acid | PFOS_INT |
| Tridecafluorohexane-1-sulfonic acid | PFHxS | [1, 2, 3, 4-13C4] Heptadecafluoro-1-octanesulfonic acid | PFOS_INT |
| Heptadecafluoro-1-octanesulfonic acid | PFOS | [1, 2, 3, 4-13C4] Heptadecafluoro-1-octanesulfonic acid | PFOS_INT |

All unlabelled PFAS were purchased from Sigma Aldrich as neat compounds with known purity. The isotopically labelled internal standard species were obtained from Wellington Laboratories as a prepared solution with a concentration of 50 µg ml^-1^ with an uncertainty of ±2.5%. For quality assurance and control an independent certified reference material (Chiron) containing all target PFAS with a concentration of 5 µg ml^-1^ with an uncertainty of ±5% was used.

**Performance data for the analytical methods**

**Table SI-4: Performance data for the analytical methods**

| **Short** | **Name** | **Soil** | | | **Water** | | |
| --- | --- | --- | --- | --- | --- | --- | --- |
|  |  | **LOD**  **ng kg^-1^** | **LOQ**  **ng kg^-1^** | **Recovery**  **%** | **LOD**  **ng kg^-1^** | **LOQ**  **ng kg^-1^** | **Recovery %** |
| **PFBA** | Perfluorobuteric acid | 0.81 | 2.47 | 101 | 0.77 | 2.31 | 100 |
| **PFPeA** | Perfluoropentanoic acid | 0.36 | 1.17 | 103 | 0.37 | 1.11 | 103 |
| **PFHxA** | Undecafluorohexanoic acid | 0.20 | 0.66 | 98 | 0.24 | 0.72 | 126 |
| **PFHpA** | Perfluoroheptanoic acid | 0.23 | 0.76 | 70 | 0.14 | 0.41 | 125 |
| **PFOA** | Perfluorooctanoic acid | 0.22 | 0.74 | 82 | 0.10 | 0.31 | 115 |
| **PFNA** | Perfluorononanoic acid | 0.55 | 1.70 | 92 | 0.07 | 0.22 | 119 |
| **PFDA** | Perfluorodecanoic acid | 0.32 | 1.05 | 94 | 0.14 | 0.43 | 85 |
| **PFBS** | Perfluorobutanesulfonic acid | 0.39 | 1.23 | 105 | 0.25 | 0.76 | 108 |
| **PFHxS** | Perfluorohexanesulfonic acid | 0.63 | 2.08 | 94 | 0.53 | 1.67 | 79 |
| **PFOS** | Perfluorooctanesulfonic acid | 0.46 | 1.46 | 91 | 0.38 | 1.23 | 108 |

**Table SI-5: Summarized PFAS contents in solid matter of all soils investigated (mean values of analyses in duplicate and standard deviations (STD)**

|  | PFAS content in solid matter in µg/kg dm | | | | | | | | | |
| --- | --- | --- | --- | --- | --- | --- | --- | --- | --- | --- |
|  | **PFBA** | **PFDA** | **PFHpA** | **PFHxA** | **PFPeA** | **PFNA** | **PFOA** | **PFBS** | **PFHxS** | **PFOS** |
| RA1  mean value | 10.25 | 400.43 | 27.87 | 28.07 | 29.10 | 40.18 | 83.45 | 3.75 | 1.28 | 640.52 |
| RA1 STD | 0.08 | 14.44 | 1.00 | 1.40 | 0.82 | 1.54 | 1.06 | 0.13 | 0.04 | 9.06 |
| RA2  mean value | 2.26 | 205.37 | 1.09 | 6.57 | n.d. | 20.24 | 25.81 | 3.40 | 1.06 | 1.85 |
| RA2 STD | 0.03 | 1.81 | 0.30 | 0.25 | n.d. | 1.77 | 0.27 | 0.00 | 0.03 | 0.04 |
| TX  mean value | 0.93 | n.d. | n.d. | 3.53 | n.d. | n.d. | 2.00 | 3.49 | 4.84 | 177.93 |
| TX STD | 0.11 | n.d. | n.d. | 0.01 | n.d. | n.d. | 0.01 | 0.13 | 0.25 | 6.44 |
| TE  mean value | 2.80 | n.d. | n.d. | 20.56 | n.d. | n.d. | 4.81 | 5.48 | 46.87 | 971.49 |
| TE STD | 0.12 | n.d. | n.d. | 1.78 | n.d. | n.d. | 0.67 | 0.07 | 3.42 | 112.72 |

**Calculation of conditions for column percolation tests**

The contact time and the flow velocity are calculated following DIN 19528 [1]:

$v=\frac{V\cdot n}{t\cdot60}$

where

*v* is the flow velocity in ml/min,

*V* is the volume of the column filled with the sample in cm^3^,

*n* is the porosity of the sample (dimensionless) and

*t* is the contact time between sample and eluent in h.

Following the German standard DIN 19528 [3] the contact time is fixed to 5 h to facilitate the comparison between different materials and column size dimensions. To meet this requirement, the flow rate is calculated for every column test.

Slight variations in contact time and flow rate may arise during the experiments due to the back pressure of the column and depending of the pumps performance regarding the stability and linearity of flow rate calibration.

**Calculation to transform measured concentration in the leachate to release**

$$X=\frac{V\cdot c}{m}$$

where

*X* is the release in mg/kg

*V* is the volume of the eluate in l

*C* is the concentration of the analyte in µg/l

*m* is the dry weight of the sample used for elution in g **Additional comparative graphs for the PFAS concentrations and accompanying parameters in the eluates of batch tests and column percolation tests**

***

***

***Figure SI-1: PFAS concentrations in eluates from column tests for sample RA1 (selected substances with concentrations well above the LOQ)***

***

***

***Figure SI-2: PFAS concentrations in eluates from column tests for sample RA2 (selected substances with concentrations well above the LOQ)***





***Figure SI-3: PFAS concentrations in eluates from column tests for sample TE (selected substances with concentrations well above the LOQ)***



***Figure SI-4: PFAS concentrations in eluates from column tests for sample TX (selected substances with concentrations well above the LOQ)***

**

**

**Figure SI-5: pH, turbidity, and TOC (mg/l) in dependence on liquid/solid ratio (L/S) of column and batch test eluates for sample RA1**

**

**

**Figure SI-6: pH, turbidity, and TOC (mg/l) in dependence on liquid/solid ratio (L/S) of column and batch test eluates for sample RA2**

**

**

**Figure SI-7: pH, turbidity, and TOC (mg/l) in dependence on liquid/solid ratio (L/S) of column and batch test eluates for sample TE**





**Figure SI-8: pH, turbidity, and TOC (mg/l) in dependence on liquid/solid ratio (L/S) of column and batch test eluates for sample TX**

**Data on the comparison of solid-liquid separation steps**

**Table SI-6: Comparison of** **solid-liquid separation steps for batch tests at L/S 2 and 10 l/kg following DIN 19529 [4] (mean values, n=2)**

**References**

[1] DIN 38414-14: 2011-08 „Determination of selected polyfluorinated compounds (PFC) in sludge, compost and soil - Method using high performance liquid chromatography and mass spectrometric detection (HPLC-MS/MS) (S 14)“

[2] DIN 38402-51: 2017-05 “German standard methods for the examination of water, waste water and sludge - General information (group A) - Part 51: Calibration of analytical methods - Linear calibration (A 51)”

[3] DIN 19528:2009-01, Elution von Feststoffen - Perkolationsverfahren zur gemeinsamen Untersuchung des Elutionsverhaltens von organischen und anorganischen Stoffen für Materialien mit einer Korngröße bis 32 mm - Grundlegende Charakterisierung mit einem ausführlichen Säulenversuch und Übereinstimmungsuntersuchung mit einem Säulenschnelltest (Leaching of solid materials - Percolation method for the joint examination of the leaching behaviour of organic and inorganic substances for materials with a particle size up to 32 mm - Basic characterization using a comprehensive column test and compliance test using a quick column test), German Institute for Standardization (Update available, issue date 2023-07)

[4] DIN 19529:2015-12, Elution von Feststoffen - Schüttelverfahren zur Untersuchung des Elutionsverhaltens von anorganischen und organischen Stoffen mit einem Wasser/Feststoff-Verhältnis von 2 l/kg (Leaching of solid materials - Batch test for the examination of the leaching behaviour of inorganic and organic substances at a liquid to solid ratio of 2 l/kg), German Institute for Standardization (Update available, issue date 2023-07)
